# Towards a comprehensive view of the herpes B virus

**DOI:** 10.3389/fimmu.2023.1281384

**Published:** 2023-11-16

**Authors:** Jiangling Lu, Yiru Long, Jianhua Sun, Likun Gong

**Affiliations:** ^1^ State Key Laboratory of Drug Research, Shanghai Institute of Materia Medica, Chinese Academy of Sciences, Shanghai, China; ^2^ University of Chinese Academy of Sciences, Beijing, China; ^3^ Zhongshan Institute for Drug Discovery, Shanghai Institute of Materia Medica, Chinese Academy of Sciences, Zhongshan, China

**Keywords:** herpes B virus, zoonotic pathogens, diagnosis, therapy, α-herpesvirus

## Abstract

Herpes B virus is a biosafety level 4 pathogen and widespread in its natural host species, macaques. Although most infected monkeys show asymptomatic or mild symptoms, human infections with this virus can cause serious neurological symptoms or fatal encephalomyelitis with a high mortality rate. Herpes B virus can be latent in the sensory ganglia of monkeys and humans, often leading to missed diagnoses. Furthermore, the herpes B virus has extensive antigen crossover with HSV, SA8, and HVP-2, causing false-positive results frequently. Timely diagnosis, along with methods with sensitivity and specificity, are urgent for research on the herpes B virus. The lack of a clear understanding of the host invasion and life cycle of the herpes B virus has led to slow progress in the development of effective vaccines and drugs. This review discusses the research progress and problems of the epidemiology of herpes B virus, detection methods and therapy, hoping to inspire further investigation into important factors associated with transmission of herpes B virus in macaques and humans, and arouse the development of effective vaccines or drugs, to promote the establishment of specific pathogen-free (SPF) monkeys and protect humans to effectively avoid herpes B virus infection.

## 1 Introduction

Macaques share about 93% genomic homology with humans and exhibit numerous disease phenotypes similar to humans, making them an important model animal for the study of human disease (1, 2). Rhesus macaques (*Macaca mulatta*) (2) and cynomolgus macaques (*Macaca fascicularis*) *(* 3) are the most commonly used experimental macaques, playing a key role in research on various infectious diseases, including human immunodeficiency virus (HIV), Zika virus (ZIKV), and severe acute respiratory syndrome coronavirus 2 (SARS-CoV-2) infection. During the SARS-CoV-2 infection outbreak, many medical companies and academic teams urgently developed anti-SARS-CoV-2 drugs and vaccines, necessitating large amounts of experimental monkeys for preclinical trials. China has a large number of macaques, which is the backbone of scientific research in non-human primates (NHP) (4). The SARS-CoV-2 was considered a zoonotic risk during the coronavirus disease 2019 (COVID-19) pandemic. In order to fight the spread of disease, as one of the main exporters of experimental monkeys, China banned wildlife trade in January 2020, and mandated the facilities where macaques are raised to be quarantined, and the export and transport of wild animals will be prohibited. The implementation of this announcement ended in May 2022. In 2004, in order to strengthen the protection and management of wild macaque colonies and habitats, China strictly restricted the hunting of wild macaques to breed experimental monkeys. Australia has also outlawed the use of wild-caught primates in medical research, and some senators called for a ban on the importation of NHP for medical studies in 2015 (5). The National Institutes of Health (NIH) predicted in 2018 that demand for rhesus macaques and marmosets will grow over the next five years, and some NIH-sponsored NHP centers are experiencing supply shortages (6). Due to the large demand for experimental monkeys in preclinical trials, the control of countries over the import and export of experimental monkeys, and the protection of wild macaque colonies and habitats, the contradiction between supply and demand will intensify. From 2018 to 2023, the sales price of experimental monkeys soared about 15 times.

Experimental monkeys that satisfy market demand, also known as specific pathogen-free (SPF) monkeys, must be tested negative for mycobacterium tuberculosis (TB), simian retrovirus D (SRV), cercopithecine herpesvirus type 1 (B virus), simian T lymphotropic virus type 1 (STLV-1) and simian immunodeficiency virus (SIV) (7). According to international standards, SPF NHP must be self-propagating. However, in contrast to the SPF mouse breeding environment, it is more challenging to generate SPF NHP colonies, due to NHP may carry many zoonotic pathogens, which is affected by the natural environment, group environment and human environment. Thus, the quality of experimental monkeys is difficult to guarantee, causing great economic losses and increasing research costs. At the same time, these microorganisms can also infect human beings, especially occupational workers (such as primate veterinarians, animal caregivers, and laboratory researchers), threatening the safety of human life.

Herpes B virus was first identified in 1932, The International Committee on Taxonomy of Viruses (ICTV) termed it Cercopithecine herpesvirus 1 (CHV-1) in 1999 (8), which was then renamed Macacine herpesvirus 1 (McHV 1) in 2008 (9), and Macacine alphaherpesvirus 1 in 2015 (10). Herpes B virus belongs to the Herpesviridae taxonomically, α-herpesvirus subfamily, herpes simplex virus genus, in the same category as the human herpes simplex virus (HSV-1, HSV-2). Macaques (such as rhesus macaques, cynomolgus macaques and pig-tailed macaques) are natural hosts of the B virus. Herpes B virus is the only one of nearly 35 identified NHP herpesviruses that is highly pathogenic in humans, with untreated cases of the virus exceeding 70% of fatalities (11).

This review focuses on summarizing the recent progress and characteristics of viral biology and clinical epidemiology of herpes B virus, and discussing the current and future of the diagnosis, prevention and treatment of herpes B virus, hoping to inspire researchers to solve the existing hazards of herpes B virus, more effectively eliminate B virus from infected groups, and help to prevent its re-infection into established SPF NHP colonies.

## 2 Biology of the herpes B virus

### 2.1 Genome of the herpes B virus

**Figure 1:**
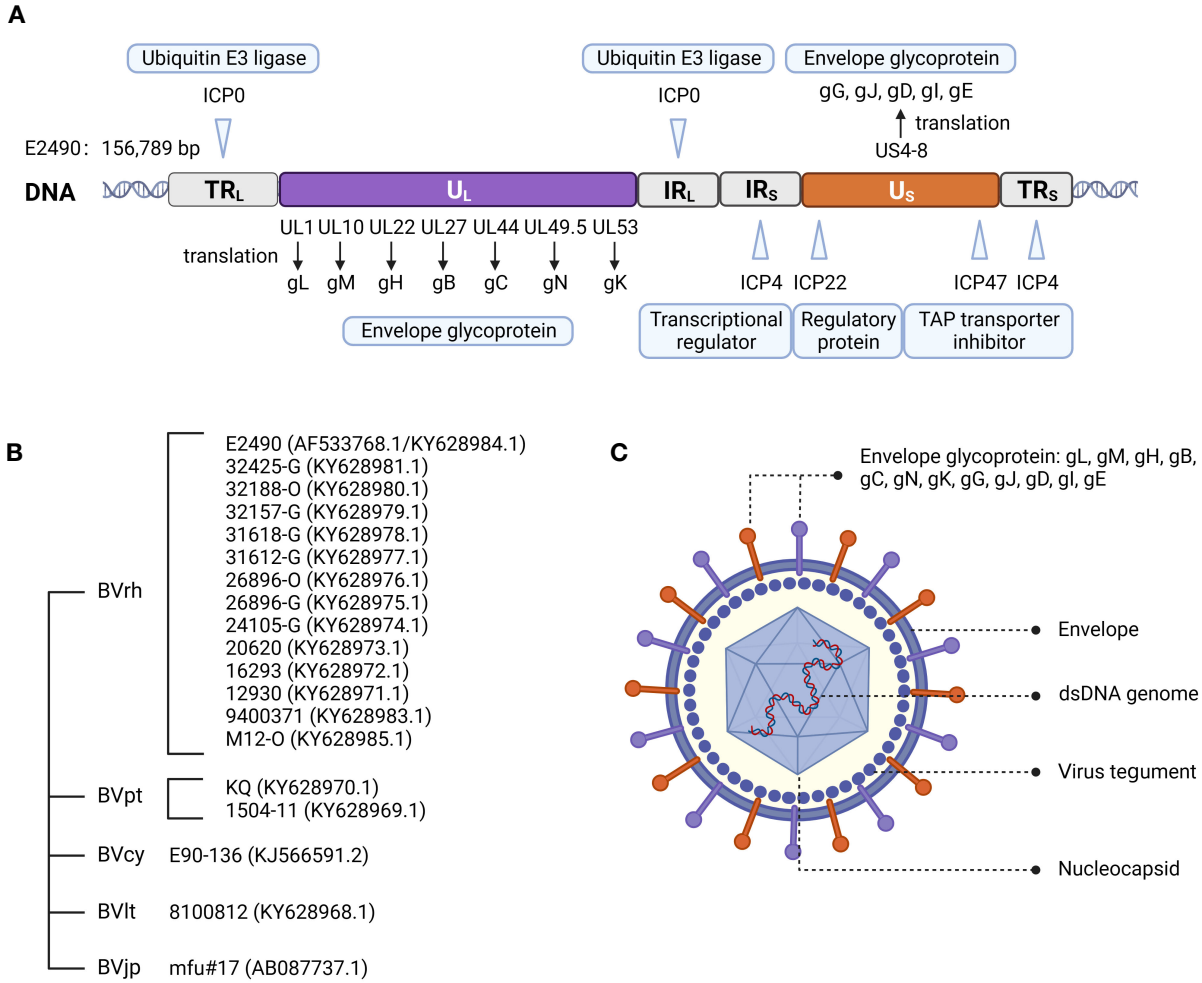
Herpes B virus genome organization, genotypes, and virion structure model. **(A)** Genome structure of the B virus. The structural organization of the B virus genome, shows the long unique region (U_L_) and the short unique region (U_S_), each bounded by an inverted internal repeat region (TR_L_, IR_L_, TR_S_, and IR_S_). The location of several functional genes (ICP0, ICP4, ICP22, ICP27 and ICP47) that impact gene expression and replication, and other genes (ul1, ul10, ul22, ul27, ul44, ul53 and US4-8) that produce envelope glycoprotein, are indicated. **(B)** Genotypes of the B virus. Sequence variation on BV isolates from different macaque species confirmed the existence of different genotypes of the B virus. These isolates from rhesus macaques (BVrh), cynomolgus macaques (BVcy), pig-tailed macaques (BVpt), lion-tailed macaques (BVlt) were completely sequenced, except for Janpanese macaques (BVjp). The complete genomes of these isolates have been sequenced, except for mfu#17. **(C)** Herpes B virus structure model.

In 2003, Perelygina et al. (12) sequenced strain E2490 (GenBank Accession: AF533768.1) isolated from rhesus macaques. The genome sequence was 156,789 bp and features the entire genome structure of α-herpesvirus (**Figure 1A**). B virus is an enveloped double-stranded DNA virus with 72 unique open reading frames (ORF). B virus share sequence homology and are co-linear with HSV-1 and HSV-2 (13, 14). All but one B virus gene were identified on the basis of sequence homology to HSV-1 and HSV-2 genes and named correspondingly. B virus genome contains two unique regions: the long unique region (U_L_) and the short unique region (U_S_). U_L_ is bounded by a terminal repeat region (TR_L_) and an inverted internal repeat region (IR_L_), and US is similarly bounded by a terminal repeat region (TR_S_) and an inverted internal repeat region (IR_S_) (8, 15). Based on the DNA buoyant density centrifugation, the content of G+C in the genome was estimated at 74.5%, second only to 76% for Simian agent 8 (SA8, also called Cercopithecine herpesvirus 2) (16), while 68.1% for HSV-1 (17), and 70.4% for HSV-2 (18).

Two major sequence differences between B virus and HSV-1 and HSV-2 genomes were detected. The B virus RS region contains an additional ≈1.5 kb of sequence between the S terminus and the ICP4 gene homolog, while RL of B virus is shorter than HSV, with no sequence homology to the ICP0 flanking region (12), and lacks homology to HSV open reading frame γ_1_34.5 (RL1), which encodes a neurovirulence factor ICP34.5 (19). Furthermore, oriL and oriS are replication origins of B virus, and are present in locations corresponding to HSV oriL and oriS locations. The nucleotide sequences of B virus oriS and oriL core elements are extremely conserved and almost identical to HSV oriL but distinct from HSV oriS (12).

Restriction endonuclease analysis showed that B virus isolates from different macaque species can be distinguished from one another. Restriction mapping of the cynomolgus macaque B virus isolate (BVcy) showed that the genome of BVcy is different from the rhesus macaque B virus isolate (BVrh) E2490 (20). Ohsawa et al. (21) reported the genome sequence of the B virus E90-136 strain (GenBank Accession: KJ566591.2) obtained from cynomolgus macaques, which was 2.9 kbp different from the BVrh genome. The majority of the divergence between this viral strain and strain E2490 can be attributed to the lack of duplication in the non-coding regions of RL and RS in BVcy. Given that these sequences have no recognized function, the relevance of this sequence variation is unclear. To date, only a few complete genome sequences of B virus in GenBank (less than twenty B virus isolates), and B virus isolated from only 5 of the 20 macaque species have been analyzed.

### 2.2 Structure of the herpes B virus

The B virus was found to be an enveloped particle with a diameter of about 200 nm, comprised of an electron dense nucleus containing viral DNA within an icosapentahedral capsid encased in an amorphous tegument protein layer, and a lipid envelope coated with viral glycoproteins (11, 15) (**Figure 1C**). Envelope glycoproteins have crucial roles in the adsorption, membrane fusion, invasion and propagation of viruses.

**Figure 2:**
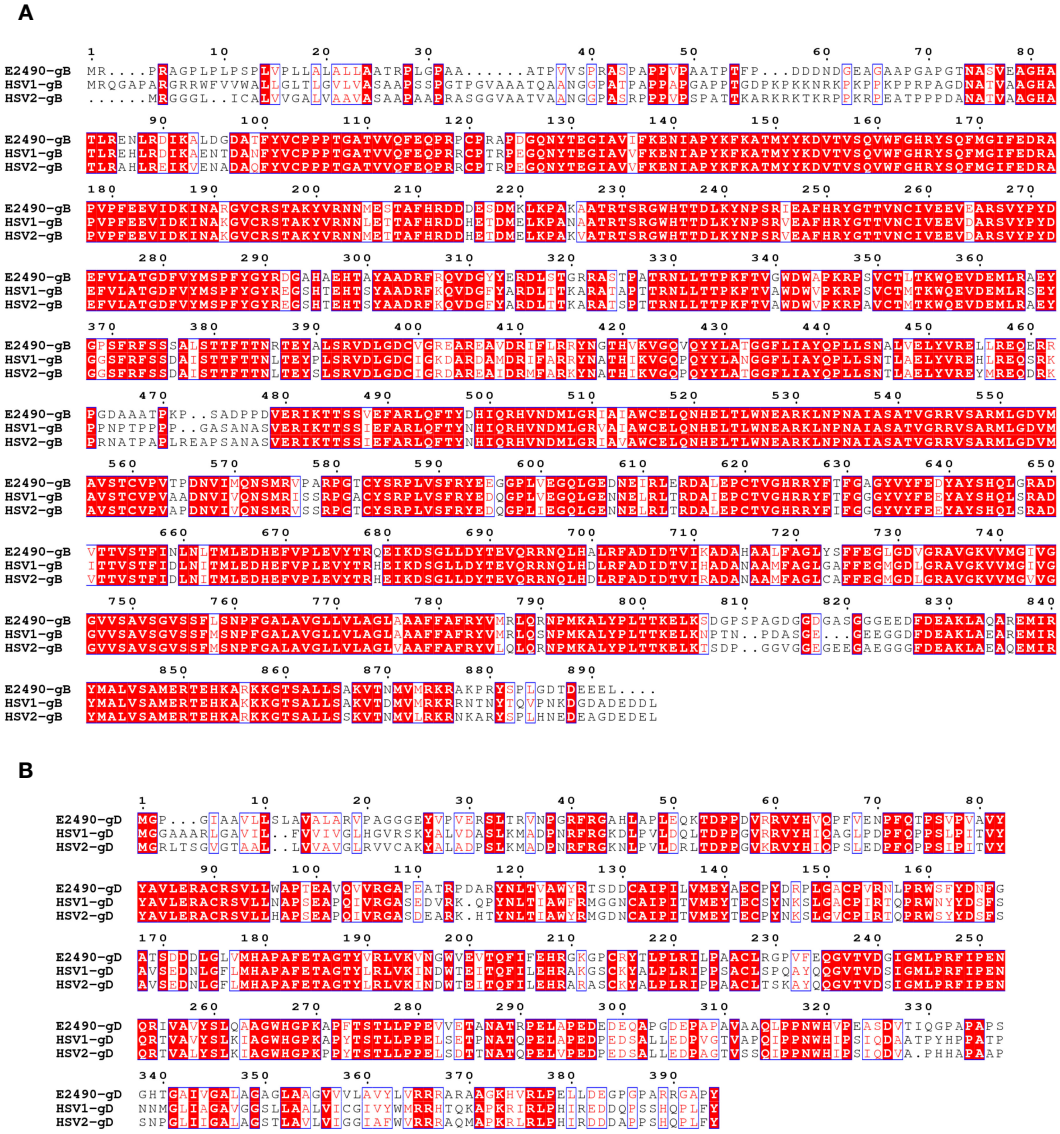
Comparison of the amino acid sequences of the gB **(A)** and gD **(B)** between B virus (E2490)and HSV (HSV-1 and HSV-2). Red box, white character: strict identity. Red character: similarity in a group. Blue frame: similarity across groups.

The degree of amino acid homology between B virus and HSV varies from 26.6% (US5) to 87.7% (UL15), and the three least-conserved proteins in B virus are US4, US5 and US12. Twelve glycoproteins are known to be present in the B virus, namely gB, gC, gD, gE, gG, gH, gI, gJ, gK, gL, gM and gN (21). Homology of the B virus to HSV-1: gB 79.9%, gD 57%, gC 49.9%, gE 46%, and gG 29.2% (12). The amino acid sequence of glycoproteins indicated that all cysteines, as well as the majority of glycosylation sites, are conserved. This conservation suggests that the glycoproteins of B virus may share a secondary structure with HSV glycoproteins (**Figure 2**).

### 2.3 Genotypes of the herpes B virus

There is no consensus on the genotyping of the B virus. Smith et al. (22) suggested that three B virus genotypes were directly related to the macaque species of origin and were composed of (I) isolates from rhesus and Japanese macaques (*Macaca fuscata*), (II) cynomolgus macaques isolates, and (III) isolates from pig-tailed macaques (*Macaca nemestrina*).

However, Tompson et al. (23) determined that the B virus derived from lion-tailed macaques (*Macaca silenus*) does not belong to the three reported genotypes and may represent the fourth genotype of the B virus. Ohsawa et al. (24) found that the B virus isolated from Japanese macaques resident in the United States and rhesus macaques had the same genotype, while the B virus isolated from native Japanese macaques and rhesus macaques were similar but not the same genotype. Eberle et al. (25) determined complete genome sequences of 19 strains of the B virus isolated from several macaque species. The re-sequenced E2490 genome (GenBank Accession: KY628984.1) and the previously published E2490 genome differed in size by 658 bp. Sequence variation between B virus isolates from different macaque species confirmed the existence of different genotypes of the B virus. Therefore, some researchers believe that herpes B virus can be composed of (I) isolates from rhesus macaques, (II) cynomolgus macaques isolates, (III) native Japanese macaques isolates, (IV) lion-tailed macaques isolates, (V) isolates from pigtail macaques (**Figure 1B**).

### 2.4 Mechanism of herpes B virus invasion into host cells

The neurotropic α-herpesvirus initiates infection in exposed mucosal tissues, and spreads rapidly to sensory and autonomic neurones, where it establishes a lifelong latency. The γ_1_34.5 gene of HSV encodes a neurotoxic factor that promotes viral replication in peripheral tissues and penetration into the peripheral nervous system (26–28), and also boosts HSV infection and replication in the central nervous system (19, 29). HSV-1 seldom causes severe encephalitis in humans, while the human infected B virus typically results in neurological symptoms and even fatal encephalomyelitis or severe neurological deficits. Although B virus shares up to 79% identity with HSV in certain amino acid segments, B virus lacks the homolog of the HSVγ_1_34.5 gene (12). It can be speculated that the B virus may utilize mechanisms different from those of HSV for efficient replication within neural cells.

Receptors, as determinants of viral tropism, are expressed in different types of cells, and their distribution limits virus infection and transmission to specific cell types. The α-herpesvirus shared entry strategies into host cells. Similar to HSV, B virus etiopathogenesis involves multifunctional viral glycoproteins combined with cellular entry receptors to execute membrane fusion. For virus entry and cell-cell fusion, herpesviruses require at least the envelope glycoproteins gB and gH/gL (30). HSV also needs the receptor-binding protein gD. The binding of gD to its corresponding receptor is a key step in viral cell entrance (31, 32). Once bound to the cell surface receptor, HSV gD undergoes an active-state conformational change, causing it to interact with gH/gL and send a signal to gB, which then undergoes a conformational change that causes it to insert into the host cell membrane to form a fusion pore and allow the virion and coat to enter the cytoplasm (33, 34). Four types of cell surface receptors bind to gD. As a receptor for HSV-1 and HSV-2 but not for other α-herpesviruses, tumor necrosis factor receptor superfamily member 14 (HVEM, also known as TNFRSF14 or CD270) is the first identified receptor for HSV entry (35), which present mainly on activated lymphocytes. The second class of receptors is Nectin-1 (36), a cell adhesion protein that serves as an entry receptor for most α-herpesviruses, and expresses on multiple tissues such as neurons, the key cells of α-herpesviruses. The third class of receptors, Nectin-2, which mediates the entry of some HSV-1 and HSV-2 strains (37, 38). 3-O-sulfonated derivatives of heparan sulfate (3-OST HS) can also act as gD-binding receptors for HSV-1 (39).

In terms of viral protein-host protein interactions the structural studies of gD of HSV suggest that gD binds to the distal membrane portion of HVEM and Nectin-1 (40–43). The binding sites of gD to HVEM are located in the N-terminal hairpin at residues 7 to 15 and 24 to 32 of gD (41). Instead, the Nectin-1 V-domain contacts a broad surface on gD formed mostly by residues from the C-terminal extension and some amino acids from the N-terminal region (40, 43). Thus, the structural comparison reveals that Nectin-1 and HVEM bind to different sites on gD, but engagement of one receptor should prevent the binding of the other (40, 41, 43–45). Paolo et al. (40) discussed the gD function of HSV-1 and concluded that residues 1-32 in the gD extracellular segment are the binding sites for HVEM, whereas residues Y38, D215, R222, and F223 bind to Nectin-1.

B virus and HSV are homologous α-herpesviruses with 57% gD identity, and studies have demonstrated that Nectin-1 can also mediate fusion between the gD of B virus and cells (46, 47). Fan et al. (46) found that B virus used human Nectin-1 as the functional entry receptor but did not use human HVEM as an entry receptor in cell-cell fusion assays. Despite the conservation of the gD amino acid residues essential for HSV-1 entry via HVEM, Patrusheva et al. (47) observed that the clinical strains of B virus were unable to use human HVEM as a receptor, and postulated that residues R7, R11 and G15 are primarily responsible for the inability of B virus to utilize HVEM for entry. They also found that a single amino acid substitution (D122N) in the IgV-core of the gD reduced the cell infectivity of human Nectin-1. The enhanced ability of B virus gD to use Nectin-2 is a feature that distinguished B virus from HSV-1 clinical strains that either were unable to utilize Nectin-2 for entry or used this receptor inefficiently. Therefore, Nectin-1 is the dominant receptor for B virus infection, and the distribution of Nectin-1 determines the similar neurotropic properties of B virus compared to HSV. Furthermore, paired immunoglobulin-like receptor α (PILRα) is an HSV-1 gB fusion receptor and mediates fusion through the O-glycosylation sites on gB. The difference in glycosylation sites between B virus and HSV-1 might be the reason why PILRα has much less fusion activity than Nectin-1 and HVEM (46). Thus, α-herpesviruses have different preferences for cell entry receptors.

Notably, Perelygina et al. (48) found that B virus gD polyclonal antibodies were ineffective to neutralize B virus infectivity on epithelial cell lines, suggesting that gD is not essential for B virus entry into these cells. In addition, they demonstrated that B viruses lacking gD could also infect human or monkey epidermal cells as efficiently as wild-type B viruses, so they believed that B virus-specific unidentified protein interactions existed to allow virus entry into host epidermal cells (49). But gD might be required for B virus entry into some targets not tested in this study [e.g., central nervous system (CNS) neurons] or for spread (e.g., retrograde or anterograde transneuronal spread). Moreover, the importance of gD is not conserved in neurophilic herpesviruses, and gD has been demonstrated in other α-herpesviruses as dispensable for intercellular spread and nerve invasion (50–52).

**Figure 3:**
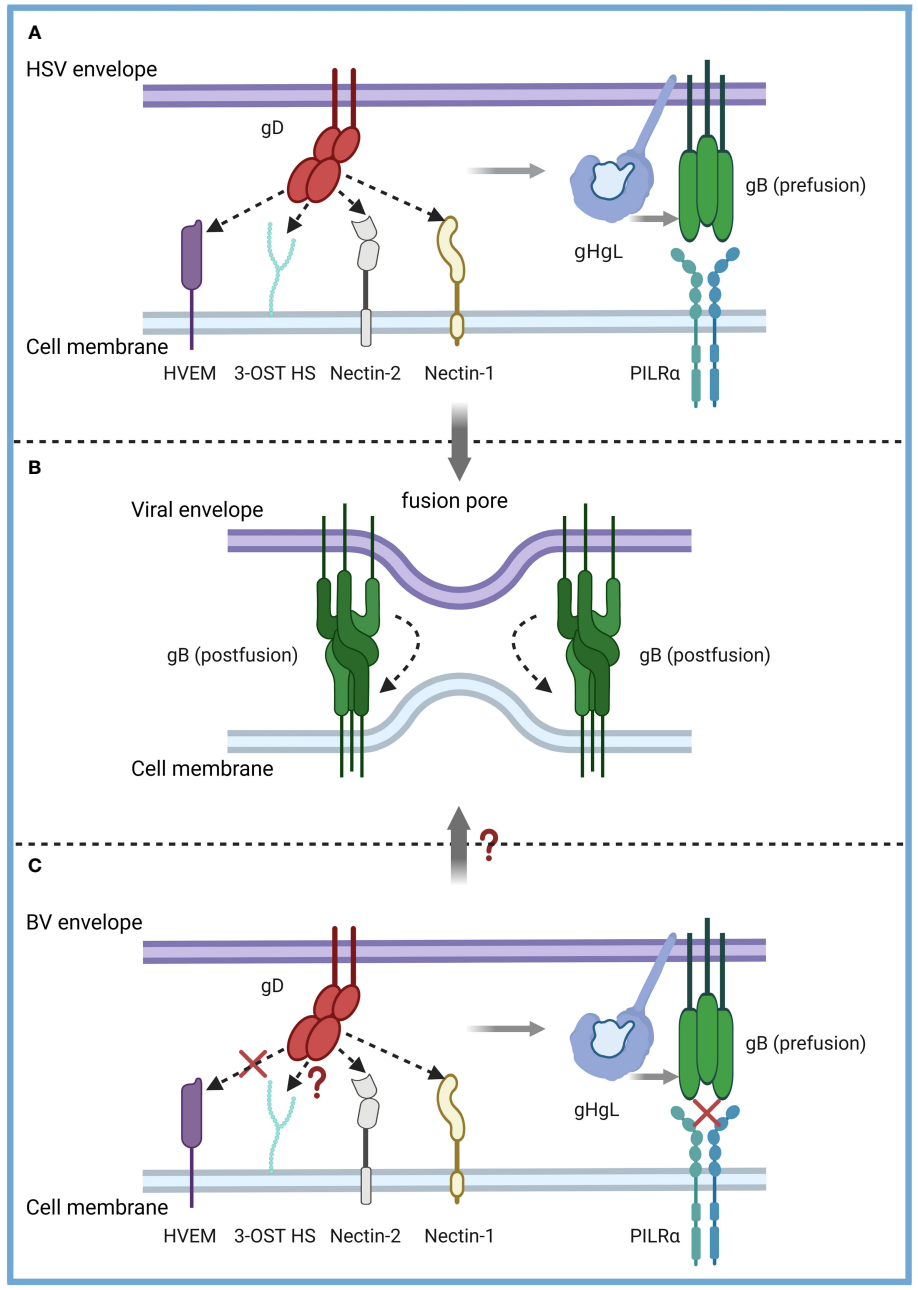
Model of the B virus and HSV entry mechanisms. α-herpesvirus shared entry strategies into host cells. **(A)** HSV-1 and HSV-2 fuse with a host cell at the cell membrane. The gD dimer (red), gH-gL heterodimer (dark and light blue), and gB trimer (green) are necessary and sufficient for entry. gD binds to several entry receptors (small arrow), including HVEM (purple), Nectin-1 (yellow), Nectin-2 (grey), and 3-OST HS (light green). The binding of gD to its corresponding receptor transmits a signal to gH-gL (light grey arrow), which activates the fusion protein gB (light grey arrow) to undergo a conformational change. **(B)** As gB refolds into its postfusion conformation, the merging of membranes forms a fusion pore through which the viral content can enter the host cells. **(C)** The entry mechanisms of the B virus and HSV are similar but not identical. B virus can effectively use both Nectin-1 and Nectin-2 as cellular receptors for entry into human cells but not the HVEM-mediated entry pathway (red fork). B virus utilizes 3-OST HS to bind gD, which is unknown (red question mark). PILRα, an HSV-1 gB fusion receptor, and did not function as an entry receptor (red fork). Exactly how B viruses achieve membrane fusion (red question mark) still needs to be studied more systematically and deeply.

Elucidating the invasion mechanism and life cycle characteristics of the B virus is the basis for its diagnosis, prevention and treatment. However, at present, we have not studied in detail the mechanism and life cycle changes of B virus into natural host or human target cells, and the most well-researched interaction between gD and Nectin-1 still lacks conclusive evidence. Existing studies have shown that B virus and HSV are similar but not identical, and our understanding of HSV can only serve to inspire the study of B virus (**Figure 3**).

## 3 Epidemiology of the herpes B virus

### 3.1 Epidemiology of B virus infection in monkeys

The B virus is carried naturally by macaques of the Old World monkeys (such as rhesus, cynomolgus and pig-tailed macaques), which are usually latent and asymptomatic or cause only mild disease. Significant lesions of the tongue and lips were found only in 332 (2.3%) of 14,400 rhesus monkeys examined by Keeble et al. (53). However, disseminated viral infection is rare in macaques and is usually fatal when it occurs (54, 55). Non-macaques (all New World monkeys and other Old World monkeys) are not at risk of infecting the B virus unless they are raised with macaques, such as DeBrazza’s monkeys (*Cercopithecus neglectus*) (23), capuchin monkeys (*Cebus apella*) (56), patas monkeys and a black and white colobus monkey (57), which may contract the B virus and eventually die.

Infection with the B virus is usually mild and self-limited in Asian macaques. Nevertheless, in some cases, diseases similar to human HSV infection may occur (e.g., vesicular lesions on the tongue and lips, sometimes on the skin). Although the presence of herpetic skin lesions means the active transmission, B virus-infected macaques without visible mucosal lesions can still transmit the virus. Some slight symptoms may subside within 10-14 days without scarring, occasionally conjunctivitis of varying severity, and no genital tract damage symptoms. But B virus infection in monkeys is lifelong, because the virus can be in the trigeminal ganglia and lumbosacral nerve for a long time (58–60). During periods of stress or immunosuppression in macaques, B virus can be reactivated and shed from the oral, nasal, or genital mucosa without signs of clinical illness (7, 61). Normally, shedding lasts several hours, but in monkeys with initial infection, secondary infection, or other disorders, shedding can last 4 to 6 weeks. The shedding probability of monkeys infected with the B virus is 1-2% (62).

B virus infection is common in macaques and the incidence rises with age. It is mainly transmitted through mating, scratching or biting. In sexually mature non-SPF rhesus macaques, the incidence of B virus infection is low, but it rapidly increased after sexual maturity at 3-4 years old, approaching 80-90% B virus-positivity in some groups (63–67). During the breeding season, adolescent macaques aged 2-3 years had the highest risk of infection (64, 65). The chance of vertical transmission from mother to baby cynomolgus macaques is rare (68). Five baby cynomolgus macaques, which were born in captivity to seropositive mothers, all had B virus antibody titers of 16 to 32 when tested within 1 month of birth, and had disappeared after about 3 months. Weigler et al. (65) found that in the B virus antibody-positive group, each tested infant monkey (n = 28) was positive with a titer equal to the mother and disappeared at an average age of 5.5 months. Therefore, it is extremely likely that the positive mother transmits the B virus antibody to the newborn macaques through the placenta, so that the newborn macaques acquire passive immunity. However, sexually immature macaques may become infected after close contact with infected mothers or other infected macaques in the group.

### 3.2 Epidemiology of B virus infection in humans

Human infections are typically caused by monkey bites or scratches, contamination injuries caused by monkey experiment supplies (e.g., needle and scalpel blade), or exposure to mucosal infectious substances (e.g., secretions of the ocular, oral or genital; feces, CNS tissue and cerebrospinal fluid) (69–71). Exposure to a macaque’s blood does not lead to exposure to the B virus, given that viremia is regarded as rare among infected macaques (72). B virus infections in people are uncommon, but the lethality of untreated patients is up to 70%. To date, all documented human B virus infections are symptomatic, and there is no serological evidence that the B virus can cause asymptomatic infection in humans. Some individuals developed symptoms within 48 hours of exposure (73), and typical symptoms at the beginning of infection generally include vesicular herpetic lesions, non-specific influenza-like illness and local lymphadenitis. B virus infection is present in the mucosa of cheek, gingiva, conjunctiva, anal and genital. The wound site is often accompanied by peripheral nerve symptoms (e.g., pain, numbness or pruritus) (74) and CNS symptoms (75). The terminal stage of B virus infection may lead to encephalitis, encephalomyelitis and other CNS disorders, and cause respiratory paralysis and death in some severe cases (74). For the rest of their lives, the majority of the survivors have moderate to severe nerve injuries.

The Centers for Disease Control and Prevention (CDC) claimed that about 50 cases of the B virus were recorded globally from 1932 to 2019, mostly caused by exposure to laboratory or captive macaques and their tissues (76). In November 2019, the first human infection of herpes B virus that occurred in Japan was reported by Tokyo Metropolitan Infectious Disease Surveillance Center (not reported in detail and the health of patient was unknown). In 2021, the Chinese Center for Disease Control and Prevention (CCDC) reported the first case of a human infected with herpes B virus died in China (77). With only one case of the human-to-human transmission, the likelihood of secondary transmission of the B virus seems minimal (78). Furthermore, the pattern of symptomatic B virus infection in at least two cases suggested that the disease was recurrent (79, 80). The level of B virus that triggers the infection in humans is unknown. B virus has not been discovered at measurable amounts in infected hosts’ blood or serum (81), but has been found in other sites such as buccal mucosa, saliva, genital fluid, or cerebrospinal fluid (82).

Moreover, the threat to humans from the B virus in global non-laboratory settings has not been studied. A perplexing feature of this zoonotic pathogen infection is that despite B virus-positive macaques having substantial-close interaction and direct exposure with humans, there were few fatal or clinically obvious B virus infections. Before the human infection with B virus in China, fatal cases of B virus have only occurred in the US and Canada following contact with captive macaques. Researchers are similarly baffled by this regional constraint of zoonotic B virus infection.

## 4 Diagnosis of the herpes B virus

### 4.1 Isolation and culture of the herpes B virus

**Figure 4:**
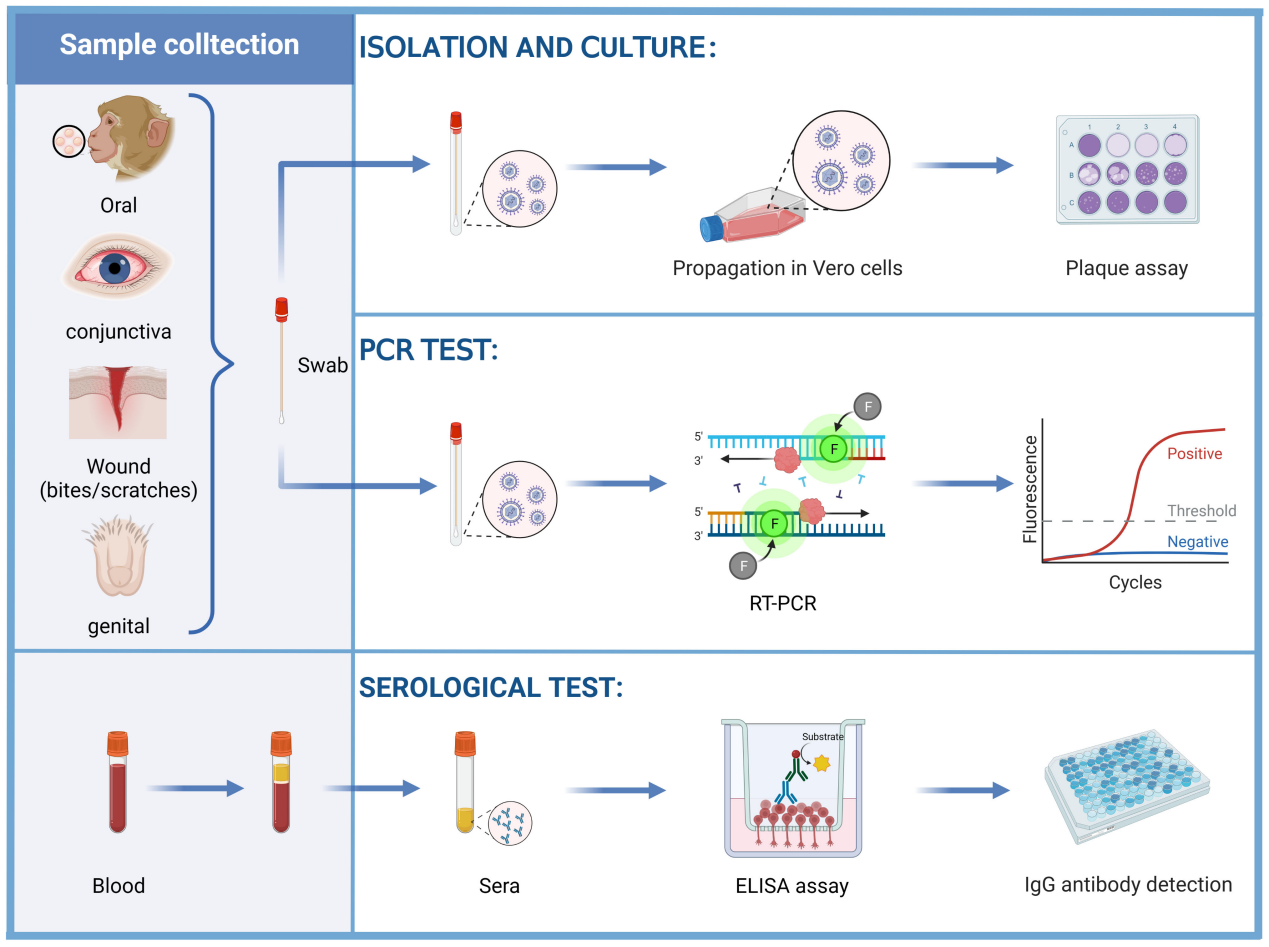
Diagnosis of the B virus. Vero cells were inoculated with swab samples obtained from the oral, conjunctiva, wound (bites and scratches), and genital epithelium, examined for CPE by plaque assay. The swab samples can also be tested for the B virus DNA by PCR tests. Herpes B virus antigen, alternative laboratory antigen, and monoclonal antibodies (mAbs) were used to coat wells of a polystyrene plate to detect anti-B virus antibodies by using ELISAs.

Among the various detection methods of the B virus, isolated culture is the standard method for the diagnosis of B virus infection. According to the CDC guidance (83), it is recommended to isolate the B virus in biosafety level 3 (BSL-3) laboratories, while expanded culture should be strictly limited to BSL-4 laboratories. At present, the cell lines that can culture B virus *in vitro* include Vero cells, Hela cells and other well-established epithelial cell lines (84–87). LLC-MK 2 cells support B virus growth rather than HSV (60), allowing these two viruses to be distinguished. The B virus was usually isolated from tissue swab specimens of the oral, conjunctiva and genital epithelium of B virus-positive monkeys. A report described the isolation of B virus from an anal swab specimen of a rhesus macaque that died of B virus infection (85). Swab specimens, cerebrospinal fluid, and perforated biopsy material from possible sites of virus inoculation (bites and scratches) in human cases of B virus infection or suspected exposure have been collected for testing, and B virus has also been detected in urine and stool specimens (78) (**Figure 4**). Detection of characteristic cytopathic effects (CPE) and eosinophilic syncytia indicates the presence of B virus (86, 88), and neutralization experiments with specific serum are required. This approach necessitates a strict operating environment and is inferior to polymerase chain reaction (PCR) tests in terms of speed, sensitivity and cost, so it does not serve as a conventional diagnostic method.

### 4.2 Nucleic acid detection

Compared to B virus culture, PCR is safer, more sensitive, more accurate, and faster. It can also be carried out under BSL-2 conditions. It is not advisable to collect wound samples for PCR testing during potential exposure because the behavior to obtain samples may push the infectious virus deeper into the wound. Instead, samples should only be taken if symptoms are consistent with B virus infection (e.g., vesicles at or near the exposed site). During active infection, B virus DNA can be detected in samples such as saliva or genitourinary samples by PCR tests (**Figure 4**).

To improve laboratory diagnosis of B virus infections, Scinicariello et al. (89) developed a PCR test by using oligonucleotide primers synthesized from the sequence of ICP 18.5 (UL 28) and probes selected for virus sequence specificity. They identified the B virus in human autopsy samples, monkey genitals and oral swabs, but not in human wound swab samples. Therefore, the fact that B virus is detected at the site of contact or in wounds does not prove B virus infection. Slomka et al. (90) developed a B virus-specific nested PCR that selected a primer pair with a relatively low GC content (60%), which located in the non-coding region between the B virus homologues of the US5 and US6 genes, and demonstrated its potential for rapid diagnosis and virus characterization. But this PCR test has only been used for cynomolgus isolates. Black et al. (91) amplified PCR products by primers were located in conserved regions of the gene encoding gB, and digested with the restriction enzyme Hae3 and electrophoresis to distinguish B virus from HSV-1, HSV-2 and HVP-2. Hirano et al. (92) added betaine to the PCR mixture to establish a PCR test to amplify a DNA segment of the gG gene of the B virus derived from a rhesus macaque. They designed primers for the gG gene of the B virus, which yielded products of 209 bp (strains from rhesus and cynomolgus macaques), 203 bp (lion-tailed macaques) and 161 bp (pig-tailed macaques), without the appearance of HSV products. This method can effectively identify B viruses and HSV. However, none of the above PCR tests is suitable for high-throughput applications because they require some post-PCR operations, such as restriction endonuclease digestion and gel electrophoresis, and none can quantify the viral load in clinical specimens. In order to understand whether the viruses are maintained in a truly latent state or one characterized by a low level of chronic expression in the natural host, Huff et al. (93) used real-time PCR to detect the samples from BVrh for the first time, and designed primers and probes for the gB gene. DNA of rhesus, cynomolgus and pig-tailed B virus field isolates was efficiently detected. In contrast, HVP-2 and HSV-2 DNA were detected inefficiently. However, the limit of detection of the assay is not known, and the assay may miss the genotype of Japanese native and lion-tailed macaques, and its sensitivity is not as good as that of ordinary PCR.

Perelygina et al. (94) designed primers and probes for the non-conserved protein gG gene of the rhesus macaque, and successfully amplified 23 B virus clinical isolates by real-time PCR, including rhesus, Japanese, cynomolgus macaques and human zoonoses. This assay took a short time (4 h), enabled high-throughput detection of samples, and distinguished B viruses from HSV-1, HSV-2, SA8, and HVP-2. Miranda et al. (95) targeted the gene of the DNA polymerase of the B virus as primers, and six of the 12 primer pairs were able to specifically detect the B virus genome and did not cross-react with the HSV-1 or HSV-2 genome. Recently, Yi Zeng et al. (96) developed a simple, rapid, sensitive and specific multiplex loop-mediated isothermal amplification (LAMP) assay for simultaneous detection of Monkeypox virus (MPXV) and B virus (primers designed for the UL 30 gene) with a limit of detection (LOD) of 28.7 and 27.8 copies per reaction, respectively. However, because there was no outbreak of MPXV and/or B viruses in China, real clinical samples that were positive for both viruses cannot be tested. Although six common human viruses were tested, including BK polyomavirus, JC polyomavirus, Cytomegalovirus, Human adenovirus type 5, HIV-1 and Hepatitis B virus, there was no comparison of HSV and other viruses highly homologous to BV. In summary, the design of PCR primers is important, requiring both identification of all genotypes and the distinction of B virus from other herpesviruses with high homology, so the conservation and specificity of target sequences should be considered when designing primers.

PCR tests can be used for quality testing in monkey colonies. Nevertheless, a significant limitation of PCR or virus isolation is that, because of the incubation period of the B virus infection, the diagnostic test would be false-negative, meaning that the presence of viral DNA could not be detected. A positive result occurs only when a latent viral infection is reactivated and the virus sheds in oral and genital secretions. Therefore, serological testing has a higher sensitivity and utility than PCR or virus isolation, which is why it is better suited for screening purposes rather than routine B virus detection. Apart from conventional PCR, real-time PCR and LAMP, digital PCR (the third-generation PCR technology) (97), PCR amplification technology combined with the CRISPR-Cas12a system (98) and microfluidic chip technology (99) have been applied to the detection of COVID-19. The effective use of these technologies in other virus detection has also provided some inspiration for the molecular diagnosis of B virus.

### 4.3 Serological diagnosis

Typically, serological diagnosis aims to detect anti-B virus antibodies, usually using enzyme-linked immunosorbent assay (ELISA), dot-immunobinding assay (DIA), and enzyme immunoassay (EIA), etc. ELISA is the standard first-line diagnostic test for B virus infection (**Figure 4**). In 1985, Heberling et al. (100) utilized inactivated homologous and heterologous antigens to ascertain the strength of the color response of the two antigens by DIA. B virus detection depends on B virus strains, but in many countries, it is difficult to obtain strains for detection. Importing B virus isolates from abroad is challenging, and BSL-4 laboratories are rare. Given that B virus, HSV-1, HSV-2, SA8 and HVP-2 have common antigens, so using a high homology and safer virus can serve as an alternative laboratory antigen to detect B virus infection without high biosafety risks. HVP-2 is genetically and more closely related to B virus than HSV-1. Ohsawa et al. (101) found that the HVP-2 antigen-based ELISA was equal in sensitivity (98%) and specificity to the BV antigen-based ELISA and was superior to the HSV-1 ELISA (96%) for the detection of BV-positive macaque sera. Takano et al. (102) used SA8 as an alternative antigen for B virus antibody detection. Seventy-two sera samples judged positive using B virus antigen were all positive when the SA8 antigen was used. Katz et al. (103) developed a high-throughput titration ELISA (tELISA) and used it to screen 278 sera simultaneously against the homologous B virus antigen and the heterologous antigens of HVP-2 and HSV-1, and B virus-ELISA detected more B virus-positive sera (35.6%) than HVP 2-ELISA (21.6%) and HSV 1-ELISA (19.8%).

In 1986, Katz et al. (104) established a rapid (3.5 h) ELISA for the detection of serum antibodies to HSV-1, SA8 and B virus. They used crude preparations of detergent-solubilized infected cells as antigens and biotinylated protein A and avidin-conjugated alkaline phosphatase as tracer reagents. In 1993, Norcott et al. (105) used a monoclonal competitive radioimmunoassay (CompRIAm) to detect anti-BV antibodies in human and monkey sera and antibodies against SA8 in monkey sera but not anti-HSV-1 antibodies in human sera. Tanabayashi et al. (106) tested gD expression in transfected COS7 cells by indirect immunofluorescence or radioimmunoprecipitation analysis (RIPA), and found that the expression of gD reacted well with BV-infected monkey sera. But this method requires the use of radioactive materials, and western blot (WB) is less reactive and appears with nonspecific bands. Subsequently, they attempted to express mutant gD as an antigen, which deleted the transmembrane domain and cytoplasmic tail, and found that the serum of positive monkeys reacted with mutant gD without nonspecific reactions by DIA. In 2005, Perelygina et al. (107) evaluated a recombinant protein-based ELISA for detecting IgG in monkey and human sera, the diagnostic sensitivity of the gB-, gC-, gD- and membrane-associated segments of gG (mgG)- ELISA was 100%, 97.3%, 88.0%, and 80.0%, respectively. And the relative diagnostic specificities of gB-, gC- and gD-ELISA were 100% and 97.5% for mgG-ELISA. The results indicate that gB, gC, gD and mgG have high diagnostic potential for serological diagnosis of B virus, while mgG may be a valuable antigen to distinguish antibodies induced by B viruses from other closely related α-herpesviruses (including HSV-1 and HSV-2), which may be related to only 29.2% identity of gG and other herpesviruses. In contrast, the sensitivity and specificity of the sgG-and gE-ELISAs are too low to be effective diagnostic antigens. In 2008, Fujima et al. (108) developed a fluorescent indirect ELISA based on recombinant gD of B virus and gG (gG-1 and gG-2, respectively) of HSV-1 and HSV-2 to distinguish the three herpesvirus infections in monkeys. In 2012, Katz et al. (109) used recombinant proteins gB, gC, gD and mgG as antigens, with higher sensitivity in BV antibody-positive sera than in WB, even in low-titer sera.

In addition to the use of envelope glycoprotein as a detection antigen, synthetic polypeptides have also been employed as an alternative antigen. Perelygina et al. (110) found that 95% of serum samples from macaques and 80% of serum samples from humans infected with B virus contained antibodies against the epitope (gD 362-370), while HSV-1 and HSV-2 antisera did not react with the epitope, so gD 362-370 had the unique potential for BV diagnosis. Hotop et al. (111) used a peptide chip with overlapping peptides covering the entire amino acid sequence of gB and gD to react with antibodies in macaque sera and identified 18 antibody target regions (ATRs), 17 of the 18 ATRs had not been described earlier. Hotop et al. (112) further performed high-throughput serology analysis based on synthetic peptide and multiplex bead flow assays of six glycoproteins (gB, gC, gD, gG, gH and gL) of B virus, and found more ATRs. However, there are issues with using peptides as the detection antigen, such as the fact that core epitopes are hard to anticipate and screen, detection sensitivity is lower than that of entire proteins, and false-negative or false-positive findings are difficult to eliminate.

Moreover, monoclonal antibodies recognize the viral antigen to detect B virus. Cropper et al. (113) prepared the monoclonal antibodies (mAbs) of B virus, HSV-1 and HSV-2, which can specifically distinguish B virus and VZV, human cytomegalovirus and EB by indirect immunofluorescent antibody test (IFAT) and distinguish cynomolgus monkey and rhesus monkey B virus strains. Blewett et al. (114) prepared mAbs targeting gB homologs. The detection of B virus by WB and RIPA showed that gB is an essential protein, but none of the anti-gB mAbs neutralized B virus. Katz et al. (115) developed a novel panel of mAbs that were used to identify specific immunoreactive epitopes of BV proteins for the first time since the discovery of this virus over 80 years ago. These mAbs identified more conformational epitopes in five B virus proteins, including gD, gI, gE, gB and VP13/14. Category-I mAbs are strain-specific and react with B virus isolates from both rhesus and Japanese macaques but not pig-tailed and cynomolgus macaques. Category-II mAbs made a differential serological diagnosis of B virus infection in humans with existing HSV-1/HSV-2 antibodies.

However, the serological diagnosis could not specifically identify the virus that was actually infected in humans or macaques. According to the positive results of B virus antibody assays, it can only be determined that the macaques were infected with B virus, but not the current status. Lees et al. (61) measured virus-specific IgM and IgG antibodies by antibody capture radioimmunoassay. IgM was first detected on day 6, while IgG did not appear until day 12 and peaked at 30 to 40 days post-infection. As with other viral infections, the presence of IgG indicates prior exposure or infection but does not indicate active virus shedding. There are few studies on the presence of B virus in serum antibody-positive animals and whether the virus is in the latent or proliferative phase (78). Furthermore, B virus-infected macaque sera tended to contain high titers of HSV-neutralizing antibodies, but human sera containing HSV antibodies failed to neutralize B virus (58). Thus, the high detection rate of HSV antibodies in humans (116) complicates the work to confirm the history of B virus infection. When B virus infects HSV immunizers, memory responses to cross-reactive antigens occur, which results in higher levels of antibodies against cross-reactive antigens, making the detection of B virus-specific antibodies more difficult. Moreover, current methods are largely based on the detection of IgG antibodies, which usually appear sometime after infection, with delays in detecting B virus infection. In addition, either false-positive, false-negative, or latent infections can complicate the interpretation of serological tests (117).

Previous studies have revealed that viral DNA is not necessarily detectable in B virus-positive macaques by PCR, and the virus is latent (81, 118), making it challenging to achieve a 100% detection rate of B virus by a single technique. In addition to combining the novel techniques mentioned above, combining molecular diagnosis and serological diagnosis can also be considered to improve the sensitivity and specificity of B virus detection. The serological diagnosis of B virus mainly depends on the level of IgG antibodies, while the more established serological diagnostic technique of SARS-Cov-2 combined detects IgA, IgM and IgG (119–121). Although IgM quickly disappears in the body, it can indicate the current infection status. And PCR tests may improve the detection rate of virus DNA of antibody-positive macaques to warn that macaques are currently active in the virus period, and occupational workers should take extra precautions to prevent B virus infection.

## 5 Prophylaxis and treatment for the herpes B virus

### 5.1 Anti-viral drugs

The relative rarity of zoonotic B virus infections does not provide a financial incentive for the development of drugs that specifically target the B virus. Therefore, the drugs used to treat HSV infection are also referred to treat patients with suspected B virus infection (**Table 1**), although B viruses are less sensitive to them than HSV (122). Neither serological nor viral testing can be used to guide the provision of antiviral prophylactic therapy. The therapeutic regimen must be based on the clinical situation. The use of anti-viral therapy early after exposure to the B virus is considered an effective strategy to prevent B virus infection in human and animal trials (78, 82, 123, 124). If a patient is suspected of being exposed to the B virus and medically evaluated within 5 days of potential exposure, post-exposure prophylaxis with high doses of oral anti-viral drugs should be considered when confirming B virus infection by diagnostic test (123, 124). Acyclovir and other nucleoside analogues are effective at high doses. For example, acyclovir 10 mg/kg q8h for 14 to 21 days. The first choice of anti-viral therapy for post-exposure prophylaxis is oral vaciclovir (suitable for adults, including pregnant women). Valaciclovir, the precursor of acyclovir, can be metabolized in the liver and intestine to acyclovir and penyclovir. The bioavailability of valacylovir is better than that of acyiclovir, manifesting as the serum acyiclovir level of metabolized valacylovir is much higher than oral acyclovir directly (75, 78). The alternative drug is high-dose oral acyclovir (75). High doses of acyclovir administered to patients within hours of exposure may prevent progression to infection or ameliorate symptomatic zoonotic B virus infection (78). If the patient develops symptoms consistent with B virus infection, post-exposure prophylaxis should be stopped and treatment for B virus infection should be initiated.

**Table 1:** The prophylaxis and treatment for B virus exposure or infection.

Clinical setting	Antiviral Therapy	Administration
Prophylaxis of B virus exposure	Valacyclovir(first choice)	1 g po q8h for 14 days
	Acyclovir	800 mg po 5 times per day for 14 days
Treatment of B virus infection		
With no CNS symptoms	Acyclovir(first choice)	12.5-15 mg/kg iv q8h
	Ganciclovir	5 mg/kg iv q12h
With CNS symptoms	Ganciclovir	5 mg/kg iv q12h

If any signs or symptoms of B virus infection or a positive B virus test result exist, intravenous anti-viral therapy is required instead of oral drugs for post-exposure prophylaxis. If CNS symptoms are present, high-dose intravenous ganciclovir is the recommended first choice (5 mg/kg q12h) (75). If CNS symptoms are absent, high-dose intravenous acyclovir is the first-line therapy for B virus infection (125). A higher intravenous dose (12.5 to 15 mg/kg q8h) is recommended for the use of acyclovir because B virus is less sensitive to acyclovir than HSV (122). In the only documented case, patients treated with ganciclovir for B virus-infected brainstem encephalitis achieved complete recovery. Although ganciclovir is more toxic than acyclovir, its potential benefit should be considered prior to administration.

In some cases, the effectiveness of human infection intervention was monitored by suppressing peripheral viral shedding and in others by reducing cerebrospinal fluid antibodies or viral DNA load (78, 82, 126). However, CNS involvement may also progress rapidly to paralysis and death, even with anti-viral therapy and supportive care. Interestingly, although it was reported that those infected with BV survived after accepting anti-viral therapy, it was also reported that medicated B virus patients died while untreated ones survived (70, 73, 78, 79, 82, 127–129). Therefore, the exact efficacy of these anti-viral drugs for B virus is not very clear, and none of these anti-viral drugs have been approved by the U.S. Food and Drug Administration (FDA) for the treatment of B virus infection.

### 5.2 Anti-serum

The observation that high-titer human anti-HSV serum neutralized B virus *in vitro* supported the idea that high-dose human γ-globulin might be useful in postexposure immunoprophylaxis against B virus infection (130, 131). Boulter et al. (132) found that simultaneous inoculation of B virus and B virus antiserum protected rabbits from lethal encephalomyelitis, and homologous (rabbit) antiserum was more effective than heterologous (monkey) antiserum. These protections apparently did not depend on the neutralization of inoculated virus but on the destruction of infected cells before they produced the progeny virus. Human immunoglobulin is readily accessible and often contains anti-HSV antibodies to neutralize B virus in heterotypic cross-reactivity, but unfortunately, the use of human γ-globulin has not been significantly effective in the limited B virus infection cases (132–134). However, Vizoso et al. found that anti-HSV antibodies could not neutralize B viruses even though there was high homology between HSV and B virus. When B virus was inoculated into VERO cells, it induced only the formation of syncytia, a CPE characteristic of this virus, after passage in the presence of antibody against HSV. Rabbits vaccinated with HSV suspension were protected from subsequent attack by B virus by a dysplastic reaction, but B virus remains latent in the dorsal root ganglia (86, 135).

### 5.3 Vaccines

Early diagnosis and treatment are effective ways to control the mortality rate of BV-infected humans. The discovery of neutralizing antibodies against B virus prompted efforts to produce B virus vaccines for use in humans or NHPs. But there is no vaccine available against B virus infection currently (**Table 2**). Sabin et al. (142) reported a failure to immunize rabbits with subclinical doses of live B virus. And ineffective attempts were made to immunize rabbits with the formalin-inactivated B virus. Rabbits survived when tested with 10 minimal infective skin doses (m.i.s.d.) of B virus, but failed to resist 100 m.i.s.d. Hull et al. (136) made a vaccine with formalin-inactivated B virus (E2490 strain) and found that rabbits producing l:4 to l:8 antibodies did resist the attack of live B virus. The vaccine could cause a mild antibody response and needed to be strengthened frequently (once in 3 months). Monkeys with pre-immune antibodies had significantly elevated neutralizing antibody titers (<1:4 or 1:16 to 1:64 or 1:128), while those without pre-immune antibodies had a maximum neutralizing antibody of 1:32, but caused bleeding after vaccination. Further studies showed that it causes heterologous anti-HSV antibody reactions *in vivo*. But the prior injection of HSV antigen does not improve rabbit anti-BV antibody levels after BV vaccine injection. Human immune responses to B virus vaccine are similar to those of rabbits, with antibody titers in the range of <1:4 to 1:16, and the use of adjuvant does not enhance the vaccination efficacy (137). These vaccines were poor immunogens, as 20% of the recipients did not respond even after repeating doses every 3 to 6 months (138). Black et al. (143) showed that neutralizing antibodies may fail to eliminate B virus *in vitro* experiments. B virus was mainly transmitted between cells but not without cells in the supernatant.

**Table 2:** Vaccines in development against the B virus.

Types of vaccines	Vaccine Components and Methodology	References
Inactivated vaccine	Formalin-inactivated B virus	Hull et al. (136–138)
Recombinant vaccine	A recombinant vaccinia virus expressing herpes B virus glycoprotein D	Bennett et al. (139)
DNA vaccine	A DNA vaccine plasmid expressing the B virus glycoprotein B	Loomis-Huff et al. (140)
	A plasmid expressing the B virus glycoprotein D	Hirano et al. (141)

With gB or gD as the immunogens (144–147), researchers developed vaccines against HSV (HSV-1 and HSV-2). The gD and gB are the main targets for neutralizing antibodies and cell-mediated immunity in patients with HSV infection. The performance of HSV vaccines is highly relevant to the development of effective vaccines for the B virus because both viruses are very similar in their respective hosts in terms of biology, pathogenicity and antigenicity. Bennett et al. (139) constructed a recombinant vaccinia virus expressing gD. Neutralizing antibody titer levels after vaccination in rabbits ranged from 1:2 to 1:8 (one exception, up to >1:64). Ten of eleven rabbits were protected within 8d and local ganglions without signs of latent B virus. Although vaccination in susceptible animal models can produce protective immune responses to B virus, it has not been reported whether this vaccination will induce protective immune responses in natural host macaques.

Loomis-Huff et al. (140) injected a DNA vaccine plasmid expressing the B virus gB into mice and rhesus macaques and caused a humoral immune response. IgGs induced by intramuscular (IM) or intradermal (ID) immunization were relatively stable over time in mice, with high titers (1:1600 to 1:25,600) maintained for up to 1 year, and all immunization methods (IM, ID, IM + ID) caused greater IgG2a responses than IgG1. The IgG2a antibody isotype predominated in the majority of immunized mice (76% and 89% at 6 and 12 weeks, respectively), suggesting of a Th1-type helper T-cell response. Rhesus macaques generated IgGs against B virus gB after IM + ID or IM immunization, with antibody titers up to 1:12,800 with neutralizing activity detected in IM + ID immunized animals. These data demonstrated that DNA immunization can be used to generate an immune response against B virus glycoproteins in uninfected macaques. Hirano et al. (141) constructed a plasmid expressing B virus gD. They injected it intrathecally into adult Japanese macaques and determined the B viral gD antibody titer via recombinant HSV-1 gD-based ELISAs. After the second booster immunization, the average titers at each time point were significantly higher than the control group, but these antibody levels did not last long. Peripheral blood mononuclear cells (PBMCs) were cultured in the presence or absence of HSV-1 gD as a stimulating antigen. Upon stimulation with the recombinant HSV-1 gD, the fraction of CD4^+^ IFN-γ^+^ T cells increased from 0.4% to 1.4%. Thus, the B virus gD DNA vaccine was shown to induce both humoral and cellular immune responses in macaques that recognize the B virus gD. Neutralizing activity in the sera of vaccinated animals was essentially the same in the presence or absence of complement.

The latent and recurrence of B virus infection have hindered the development of vaccines, and the research of the B virus vaccines has not made breakthroughs in recent years. Elimination of the B virus from the experimental macaque colonies requires preventing the initial establishment of potential infections that may subsequently reactivate. It appears from earlier research that the macaque colonies have not yet succeeded in achieving this goal. Although DNA vaccines were studied after the inactivated B virus vaccine, DNA vaccine-induced antibodies diminish relatively quickly Furthermore, studies on the pathogenesis of HSV in humans suggest that cell-mediated immunity is necessary for vaccines to provide protection or reduce the frequency of disease recurrence (148, 149). HSV-1 has been shown to have mechanisms that impede CD8^+^ T cell-mediated eradication of the virus from latency (150). Additionally, the density of CD8^+^ T cells in the genital mucosa is predictive of the duration and severity of viral reactivation (151). Despite the fact that DNA vaccines induce strong cellular immune responses theoretically, there are few studies evaluating the efficacy of B virus DNA vaccines on CD4^+^ and CD8^+^ T lymphocytes. In addition to inactivated virus vaccines and DNA vaccines, live-attenuated vaccines (152, 153), subunit vaccines (147, 154), and replication-defective virus vaccines (146, 155) have been applied to HSV, and some vaccines are already in clinical trials. While live-attenuated vaccines are particularly effective in provoking humoral and cell-mediated immune responses, one of the major challenges of live-attenuated vaccines remains vaccine safety. Subunit vaccination is safer than live-attenuated vaccine, but the challenge of developing this vaccine is to elicit a robust and durable immune response. Although the safety of replication-deficient vaccines has been demonstrated, only a few genes (gD, UL29, etc.) are knocked out, and the ramifications would be unthinkable if genetic recombination were to restore the capacity of wild virus to replicate. In addition, mRNA vaccines have also attracted the attention of many researchers due to the simplicity of mRNA design and manufacturing, inherent immunogenicity, rapid mass production and negligible insertional mutation, and have achieved great success in the development of the SARS-Cov-2 vaccine (156, 157). Some of the vaccines mentioned above are formulated as lipid nanoparticles (156), while others may use viral vector delivery technologies such as lentiviral vectors (147) or adenovirus vectors (158). There are now additional possibilities for the B virus vaccine thanks to the introduction of these new technologies. Development of B virus vaccines can be learned from the successful experiences of HSV vaccines, but the research and development of vaccines may still to be arduous and time-consuming due to the paucity of vaccine research on B virus and the obscurity of its invasion mechanism and life cycle characteristics.

## 6 Conclusion and perspectives

NHPs have become indispensable animal models in biomedical research due to their similarities in development, physiology and evolutionary relationship with humans, and the pathogens they carry have also become the focus of research. The great differences in clinical manifestations between macaques and humans infected with B virus, as well as the high mortality rate of human infection, have attracted great attention to being listed as a BSL-4 pathogen, which limits the research on B virus. But the research has never ceased, and the understanding of B virus will continue to deepen and improve. In 1989, the National Center for Research Resources [now Office of Research Infrastructure Programs (ORIP)] of the National Institutes of Health initiated experimental research contracts to establish and maintain SPF colonies, so far, many countries have built monkey colonies free of the B virus, but non-SPF monkeys are still widely used for research. Latent infection and intermittent reactivation of B virus make it quite difficult to detect infected monkeys accurately, and the antigen crossover of B virus and other pathogens also increases the difficulty of confirming B virus infection. These suggest that it is impossible to completely eliminate the possibility of B virus infection in humans.

In addition, there is no vaccine or specific neutralizing antibody drug available to prevent B virus infections. Although acyclovir or ganciclovir can be effective against the B virus, they cannot eradicate it entirely. In accordance with the standardized and secure operating procedures for experiments, therefore, prevention and fortification of personal protection are of paramount importance. When bitten or scratched by a monkey, the wound should be handled immediately, detected and treated in time, even if it is an SPF monkey. It is not possible to take chances, even if the number of individuals infected with the B virus is far lower than the number of persons scratched and bitten by monkeys. Since the B virus may remain dormant in the human body, false-negative tests may conceal serious health risks.

The biological characteristics of the B virus have been relatively well studied, but the pathogenesis and immune response to the B virus in macaques and humans need to be further studied. The specific diagnostic methods, medications and vaccines also require deeper investigation and development. Only by preventing well, establishing rapid and accurate diagnostic methods, and developing efficient and feasible treatment methods can it be possible to eliminate the risk of human mortality caused by herpes B virus infection.

## Author contributions

JL: Writing – original draft, Writing – review & editing. YL: Writing – review & editing. JS: Funding acquisition, Writing – review & editing. LG: Writing – review & editing.

## References

[1] Boffelli D McAuliffe J Ovcharenko D Lewis KD Ovcharenko I Pachter L. Phylogenetic shadowing of primate sequences to find functional regions of the human genome. Science (2003) 299(5611):1391–4. doi: 10.1126/science.1081331 12610304

[2] Rhesus Macaque Genome S Analysis C Gibbs RA Rogers J Katze MG Bumgarner R. Evolutionary and biomedical insights from the rhesus macaque genome. Science (2007) 316(5822):222–34. doi: 10.1126/science.1139247 17431167

[3] Bolton ID. Chapter 5 - basic physiology of Macaca fascicularis. In: Bluemel J Korte S Schenck E Weinbauer GF, editors. The Nonhuman Primate in Nonclinical Drug Development and Safety Assessment. San Diego: Academic Press (2015). p. 67–86.

[4] Cyranoski D. Monkey kingdom. Nature (2016) 532(7599):300–2. doi: 10.1038/532300a 27111614

[5] Nogrady B. Proposal to ban imported monkeys catches scientists off guard. Nature (2016) 530(7591):394. doi: 10.1038/nature.2016.19419 26911759

[6] Alan J Feister AD Yuenger J Ireland K Rao A. Nonhuman Primate Evaluation and Analysis Part 1: Analysis of Future Demand and Supply. National Institutes of H (2018). Available at: https://orip.nih.gov/sites/default/files/508%20NHP%20Evaluation%20and%20Analysis%20Final%20Report%20-%20Part%201%20Update%2030Oct2018_508.pdf.

[7] Morton WR Agy MB Capuano SV Grant RF. Specific pathogen-free macaques: definition, history, and current production. ILAR J (2008) 49(2):137–44. doi: 10.1093/ilar.49.2.137 18323576

[8] Weigler BJ. Biology of B virus in macaque and human hosts: A review. Clin Infect Dis an Off Publ Infect Dis Soc America (1992) 14(2):555–67. doi: 10.1093/clinids/14.2.555 1313312

[9] Davison AJ Eberle R Ehlers B Hayward GS McGeoch DJ Minson AC. The order herpesvirales. Arch Virol (2009) 154(1):171–7. doi: 10.1007/s00705-008-0278-4 PMC3552636 19066710

[10] Eberle R Jones-Engel L. Questioning the extreme neurovirulence of monkey B virus (Macacine alphaherpesvirus 1). Adv Virol (2018) 2018:5248420. doi: 10.1155/2018/5248420 29666644 PMC5831965

[11] Huff JL Barry PA. B-virus (Cercopithecine herpesvirus 1) infection in humans and macaques: potential for zoonotic disease. Emerg Infect Dis (2003) 9(2):246–50. doi: 10.3201/eid0902.020272 PMC2901951 12603998

[12] Perelygina L Zhu L Zurkuhlen H Mills R Borodovsky M Hilliard JK. Complete sequence and comparative analysis of the genome of herpes B virus (Cercopithecine herpesvirus 1) from a rhesus monkey. J Virol (2003) 77(11):6167–77. doi: 10.1128/jvi.77.11.6167-6177.2003 PMC155011 12743273

[13] Ohsawa K Black DH Sato H Rogers K Eberle R. Sequence and genetic arrangement of the ul region of the monkey B virus (Cercopithecine herpesvirus 1) genome and comparison with the Ul region of other primate herpesviruses. Arch Virol (2003) 148(5):989–97. doi: 10.1007/s00705-003-0011-2 12721804

[14] Ohsawa K Black DH Sato H Eberle R. Sequence and genetic arrangement of the U(S) region of the monkey B virus (Cercopithecine herpesvirus 1) genome and comparison with the U(S) regions of other primate herpesviruses. J Virol (2002) 76(3):1516–20. doi: 10.1128/jvi.76.3.1516-1520.2002 PMC135856 11773425

[15] Hu G Du H Liu Y Wu G Han J. Herpes B virus: history, zoonotic potential, and public health implications. Biosafety Health (2022) 4(4):213–9. doi: 10.1016/j.bsheal.2022.05.005

[16] Tyler SD Peters GA Severini A. Complete genome sequence of cercopithecine herpesvirus 2 (Sa8) and comparison with other simplexviruses. Virology (2005) 331(2):429–40. doi: 10.1016/j.virol.2004.09.042 15629785

[17] McGeoch DJ Dolan A Donald S Brauer DH. Complete DNA sequence of the short repeat region in the genome of herpes simplex virus type 1. Nucleic Acids Res (1986) 14(4):1727–45. doi: 10.1093/nar/14.4.1727 PMC339569 3005980

[18] Dolan A Jamieson FE Cunningham C Barnett BC McGeoch DJ. The genome sequence of herpes simplex virus type 2. J Virol (1998) 72(3):2010–21. doi: 10.1128/jvi.72.3.2010-2021.1998 PMC109494 9499055

[19] Chou J Kern ER Whitley RJ Roizman B. Mapping of herpes simplex virus-1 neurovirulence to gamma 134.5, a gene nonessential for growth in culture. Science (1990) 250(4985):1262–6. doi: 10.1126/science.2173860 2173860

[20] Wall LV Zwartouw HT Kelly DC. Discrimination between twenty isolates of herpesvirus simiae (B virus) by restriction enzyme analysis of the viral genome. Virus Res (1989) 12(3):283–96. doi: 10.1016/0168-1702(89)90044-0 2543157

[21] Ohsawa K Black D Ohsawa M Eberle R. Genome sequence of a pathogenic isolate of monkey B virus (Species macacine herpesvirus 1). Arch Virol (2014) 159(10):2819–21. doi: 10.1007/s00705-014-2130-3 PMC4175994 24903602

[22] Smith AL Black DH Eberle R. Molecular evidence for distinct genotypes of monkey B virus (Herpesvirus simiae) which are related to the macaque host species. J Virol (1998) 72(11):9224–32. doi: 10.1128/jvi.72.11.9224-9232.1998 PMC110342 9765470

[23] Thompson SA Hilliard JK Kittel D Lipper S Giddens WE Jr. Black DH. Retrospective analysis of an outbreak of B virus infection in a colony of Debrazza's monkeys (Cercopithecus neglectus). Comp Med (2000) 50(6):649–57. 11200573

[24] Ohsawa K Black DH Torii R Sato H Eberle R. Detection of a unique genotype of monkey B virus (Cercopithecine herpesvirus 1) indigenous to native Japanese macaques (Macaca fuscata). Comp Med (2002) 52(6):555–9. 12540170

[25] Eberle R Maxwell LK Nicholson S Black D Jones-Engel L. Genome sequence variation among isolates of monkey B virus (Macacine alphaherpesvirus 1) from captive macaques. Virology (2017) 508:26–35. doi: 10.1016/j.virol.2017.05.001 28494342 PMC5535784

[26] Whitley RJ Kern ER Chatterjee S Chou J Roizman B. Replication, establishment of latency, and induced reactivation of herpes simplex virus gamma 1 34.5 deletion mutants in rodent models. J Clin Invest (1993) 91(6):2837–43. doi: 10.1172/JCI116527 PMC443352 8390490

[27] Perng GC Ghiasi H Slanina SM Nesburn AB Wechsler SL. High-dose ocular infection with a herpes simplex virus type 1 Icp34.5 deletion mutant produces no corneal disease or neurovirulence yet results in wild-type levels of spontaneous reactivation. J Virol (1996) 70(5):2883–93. doi: 10.1128/jvi.70.5.2883-2893.1996 PMC190146 8627763

[28] Mao H Rosenthal KS. Strain-dependent structural variants of herpes simplex virus type 1 Icp34.5 determine viral plaque size, efficiency of glycoprotein processing, and viral release and neuroinvasive disease potential. J Virol (2003) 77(6):3409–17. doi: 10.1128/jvi.77.6.3409-3417.2003 PMC149531 12610116

[29] MacLean AR ul-Fareed M Robertson L Harland J Brown SM. Herpes simplex virus type 1 deletion variants 1714 and 1716 pinpoint neurovirulence-related sequences in Glasgow strain 17+ between immediate early gene 1 and the 'a' Sequence. J Gen Virol (1991) 72(Pt 3):631–9. doi: 10.1099/0022-1317-72-3-631 1848598

[30] Atanasiu D Saw WT Cohen GH Eisenberg RJ. Cascade of events governing cell-cell fusion induced by herpes simplex virus glycoproteins Gd, Gh/Gl, and Gb. J Virol (2010) 84(23):12292–9. doi: 10.1128/jvi.01700-10 PMC2976417 20861251

[31] Shukla D Dal Canto MC Rowe CL Spear PG. Striking similarity of murine nectin-1alpha to human nectin-1alpha (Hvec) in sequence and activity as a glycoprotein D receptor for alphaherpesvirus entry. J Virol (2000) 74(24):11773–81. doi: 10.1128/jvi.74.24.11773-11781.2000 PMC112460 11090177

[32] Menotti L Lopez M Avitabile E Stefan A Cocchi F Adelaide J. The murine homolog of human nectin1delta serves as a species nonspecific mediator for entry of human and animal alpha herpesviruses in a pathway independent of a detectable binding to Gd. Proc Natl Acad Sci United States America (2000) 97(9):4867–72. doi: 10.1073/pnas.97.9.4867 PMC18324 10781093

[33] Cocchi F Fusco D Menotti L Gianni T Eisenberg RJ Cohen GH. The soluble ectodomain of herpes simplex virus Gd contains a membrane-proximal pro-fusion domain and suffices to mediate virus entry. Proc Natl Acad Sci United States America (2004) 101(19):7445–50. doi: 10.1073/pnas.0401883101 PMC409938 15123804

[34] Krummenacher C Supekar VM Whitbeck JC Lazear E Connolly SA Eisenberg RJ. Structure of unliganded Hsv Gd reveals a mechanism for receptor-mediated activation of virus entry. EMBO J (2005) 24(23):4144–53. doi: 10.1038/sj.emboj.7600875 PMC1356314 16292345

[35] Montgomery RI Warner MS Lum BJ Spear PG. Herpes simplex virus-1 entry into cells mediated by a novel member of the Tnf/Ngf receptor family. Cell (1996) 87(3):427–36. doi: 10.1016/s0092-8674(00)81363-x 8898196

[36] Geraghty RJ Krummenacher C Cohen GH Eisenberg RJ Spear PG. Entry of alphaherpesviruses mediated by poliovirus receptor-related protein 1 and poliovirus receptor. Science (1998) 280(5369):1618–20. doi: 10.1126/science.280.5369.1618 9616127

[37] Krummenacher C Baribaud F Ponce de Leon M Baribaud I Whitbeck JC Xu R. Comparative usage of herpesvirus entry mediator a and nectin-1 by laboratory strains and clinical isolates of herpes simplex virus. Virology (2004) 322(2):286–99. doi: 10.1016/j.virol.2004.02.005 15110526

[38] Warner MS Geraghty RJ Martinez WM Montgomery RI Whitbeck JC Xu R. A cell surface protein with herpesvirus entry activity (Hveb) confers susceptibility to infection by mutants of herpes simplex virus type 1, herpes simplex virus type 2, and pseudorabies virus. Virology (1998) 246(1):179–89. doi: 10.1006/viro.1998.9218 9657005

[39] Shukla D Liu J Blaiklock P Shworak NW Bai X Esko JD. A novel role for 3-O-sulfated heparan sulfate in herpes simplex virus 1 entry. Cell (1999) 99(1):13–22. doi: 10.1016/s0092-8674(00)80058-6 10520990

[40] Di Giovine P Settembre EC Bhargava AK Luftig MA Lou H Cohen GH. Structure of herpes simplex virus glycoprotein D bound to the human receptor nectin-1. PloS Pathog (2011) 7(9):e1002277. doi: 10.1371/journal.ppat.1002277 21980294 PMC3182920

[41] Carfi A Willis SH Whitbeck JC Krummenacher C Cohen GH Eisenberg RJ. Herpes simplex virus glycoprotein D bound to the human receptor Hvea. Molecular Cell (2001) 8 1:169–79. doi: 10.1016/S1097-2765(01)00298-2 11511370

[42] Li A Lu G Qi J Wu L Tian K Luo T. Structural basis of nectin-1 recognition by pseudorabies virus glycoprotein D. PloS Pathog (2017) 13(5):e1006314. doi: 10.1371/journal.ppat.1006314 28542478 PMC5453625

[43] Lu G Zhang N Qi J Li Y Chen Z Zheng C. Crystal structure of herpes simplex virus 2 Gd bound to nectin-1 reveals a conserved mode of receptor recognition. J Virol (2014) 88(23):13678–88. doi: 10.1128/jvi.01906-14 PMC4248990 25231300

[44] Krummenacher C Nicola AV Whitbeck JC Lou H Hou W Lambris JD. Herpes simplex virus glycoprotein D can bind to poliovirus receptor-related protein 1 or herpesvirus entry mediator, two structurally unrelated mediators of virus entry. J Virol (1998) 72(9):7064–74. doi: 10.1128/jvi.72.9.7064-7074.1998 PMC109927 9696799

[45] Whitbeck JC Muggeridge MI Rux AH Hou W Krummenacher C Lou H. The major neutralizing antigenic site on herpes simplex virus glycoprotein D overlaps a receptor-binding domain. J Virol (1999) 73(12):9879–90. doi: 10.1128/jvi.73.12.9879-9890.1999 PMC113037 10559300

[46] Fan Q Amen M Harden M Severini A Griffiths A Longnecker R. Herpes B virus utilizes human nectin-1 but not Hvem or pilralpha for cell-cell fusion and virus entry. J Virol (2012) 86(8):4468–76. doi: 10.1128/JVI.00041-12 PMC3318624 22345445

[47] Patrusheva I Perelygina L Torshin I LeCher J Hilliard J. B virus (Macacine herpesvirus 1) divergence: variations in glycoprotein D from clinical and laboratory isolates diversify virus entry strategies. J Virol (2016) 90(20):9420–32. doi: 10.1128/jvi.00799-16 PMC5044838 27512063

[48] Perelygina L Patrusheva I Zurkuhlen H Hilliard JK. Characterization of B virus glycoprotein antibodies induced by DNA immunization. Arch Virol (2002) 147(11):2057–73. doi: 10.1007/s00705-002-0889-0 12417944

[49] Perelygina L Patrusheva I Vasireddi M Brock N Hilliard J. B Virus (Macacine Herpesvirus 1) Glycoprotein D Is Functional but Dispensable for Virus Entry into Macaque and Human Skin Cells. J Virol (2015) 89(10):5515–24. doi: 10.1128/JVI.03568-14 PMC4442516 25740986

[50] Ch'ng TH Spear PG Struyf F Enquist LW. Glycoprotein D-independent spread of pseudorabies virus infection in cultured peripheral nervous system neurons in a compartmented system. J Virol (2007) 81(19):10742–57. doi: 10.1128/jvi.00981-07 PMC2045490 17652377

[51] Peeters B de Wind N Hooisma M Wagenaar F Gielkens A Moormann R. Pseudorabies virus envelope glycoproteins Gp50 and Gii are essential for virus penetration, but only Gii is involved in membrane fusion. J Virol (1992) 66(2):894–905. doi: 10.1128/jvi.66.2.894-905.1992 1309919 PMC240790

[52] Suenaga T Satoh T Somboonthum P Kawaguchi Y Mori Y Arase H. Myelin-associated glycoprotein mediates membrane fusion and entry of neurotropic herpesviruses. Proc Natl Acad Sci United States America (2010) 107(2):866–71. doi: 10.1073/pnas.0913351107 PMC2818916 20080767

[53] Keeble SA. B virus infection in monkeys. Ann New York Acad Sci (1960) 85:960–9. doi: 10.1111/j.1749-6632.1960.tb50016.x 13752136

[54] Pöhlmann S Suntz M Akimkin V Bleyer M Kaul AJ. Herpes B virus replication and viral lesions in the liver of a cynomolgus macaque which died from severe disease with rapid onset. J Med Primatol (2017) 46:256–9. doi: 10.1111/jmp.12269 28439900

[55] Carlson CS O'Sullivan MG Jayo MJ Anderson DK Harber ES Jerome WG. Fatal disseminated cercopithecine herpesvirus 1 (Herpes B infection in cynomolgus monkeys (Macaca fascicularis). Vet Pathol (1997) 34(5):405–14. doi: 10.1177/030098589703400504 9381651

[56] Coulibaly C Hack R Seidl J Chudy M Itter G Plesker R. A natural asymptomatic herpes B virus infection in a colony of laboratory brown capuchin monkeys (Cebus apella). Lab Anim (2004) 38(4):432–8. doi: 10.1258/0023677041958891 15479559

[57] Loomis MR O'Neill T Bush M Montali RJ. Fatal herpesvirus infection in Patas monkeys and a black and white colobus monkey. J Am Vet Med Assoc (1981) 179(11):1236–9. 6276349

[58] Vizoso AD. Recovery of herpes simiae (B virus) from both primary and latent infections in rhesus monkeys. Br J Exp Pathol (1975) 56(6):485–8. PMC2072792 177038

[59] Boulter EA. The isolation of monkey B virus (Herpesvirus simiae) from the trigeminal ganglia of a healthy seropositive rhesus monkey. J Biol Standardization (1975) 3(3):279–80. doi: 10.1016/0092-1157(75)90031-1 169266

[60] Zwartouw HT. Excretion of B virus in monkeys and evidence of genital infection. Lab Anim (1984) 18(no. 1):65–70-1984. doi: 10.1258/002367784780864929 10628791

[61] Lees DN Baskerville A Cropper LM Brown DW. Herpesvirus simiae (B virus) antibody response and virus shedding in experimental primary infection of cynomolgus monkeys. Lab Anim Sci (1991) 41(4):360–4. 1658484

[62] Jones-Engel L Engel GA Heidrich J Chalise M Poudel N Viscidi R. Temple monkeys and health implications of commensalism, Kathmandu, Nepal. Emerg Infect Dis (2006) 12(6):900–6. doi: 10.3201/eid1206.060030 PMC3373059 16707044

[63] Kessler MJ Hilliard JK. Seroprevalence of B virus (Herpesvirus simiae) antibodies in a naturally formed group of rhesus macaques. J Med Primatol (1990) 19(2):155–60. doi: 10.1111/j.1600-0684.1990.tb00422.x 2160017

[64] Sariol CA González-Martínez J Arana T Gascot S Suárez E Maldonado E. Differential distribution of antibodies to different viruses in young animals in the free-ranging Rhesus Macaques of Cayo Santiago. J Med Primatol (2006) 35(6):369–75. doi: 10.1111/j.1600-0684.2006.00174.x 17214665

[65] Weigler BJ Hird DW Hilliard JK Lerche NW Roberts JA Scott LM. Epidemiology of cercopithecine herpesvirus 1 (B virus) infection and shedding in a large breeding cohort of rhesus macaques. J Infect Dis (1993) 167(2):257–63. doi: 10.1093/infdis/167.2.257 8380607

[66] Wisely SM Sayler KA Anderson CJ Boyce CL Klegarth AR Johnson SA. Macacine herpesvirus 1 antibody prevalence and DNA shedding among invasive rhesus macaques, Silver Springs State Park, Florida, USA. Emerg Infect Dis (2018) 24(2):345–51. doi: 10.3201/eid2402.171439 PMC5782895 29350146

[67] Lee MH Rostal MK Hughes T Sitam F Lee CY Japning J. Macacine herpesvirus 1 in long-tailed Macaques, Malaysia, 2009-2011. Emerg Infect Dis (2015) 21(7):1107–13. doi: 10.3201/eid2107.140162 PMC4480374 26080081

[68] Zwartouw HT MacArthur JA Boulter EA Seamer JH Marston JH Chamove AS. Transmission of B virus infection between monkeys especially in relation to breeding colonies. Lab Anim (1984) 18(2):125–30. doi: 10.1258/002367784780891352 6087022

[69] Control CfD, Prevention. B Virus (Herpes B, Monkey B Virus, Herpesvirus Simiae, and Herpesvirus B). (2012). Available at: https://www.cdc.gov/herpesbvirus/index.html.

[70] Centers for Disease C, Prevention. Fatal cercopithecine herpesvirus 1 (B virus) infection following a mucocutaneous exposure and interim recommendations for worker protection. MMWR Morbid Mortal Wkly Rep (1998) 47(49):1073–6. 9879633

[71] Freifeld AG Hilliard J Southers J Murray M Savarese B Schmitt JM. A controlled seroprevalence survey of primate handlers for evidence of asymptomatic herpes B virus infection. J Infect Dis (1995) 171(4):1031–4. doi: 10.1093/infdis/171.4.1031 7706783

[72] Hilliard J ed. Strategies of managing macaque monkeys and Herpesvirus simiae (B virus). In: Proceedings of the 4th National Symposium on Biosafety: Working Safely with Research Animals. Available at: https://search.library.wisc.edu/catalog/999888698302121.

[73] Davidson WL Hummeler K. B virus infection in man. Ann New York Acad Sci (1960) 85:970–9. doi: 10.1111/j.1749-6632.1960.tb50017.x 13720072

[74] Elmore D Eberle R. Monkey B virus (Cercopithecine herpesvirus 1). Comp Med (2008) 58(1):11–21. 19793452 PMC2703160

[75] Cohen JI Davenport DS Stewart JA Deitchman S Hilliard JK Chapman LE. Recommendations for prevention of and therapy for exposure to B virus (Cercopithecine herpesvirus 1). Clin Infect Dis an Off Publ Infect Dis Soc America (2002) 35(10):1191–203. doi: 10.1086/344754 12410479

[76] Prevention CfDCa. B Virus (Herpes B, Monkey B Virus, Herpesvirus Simiae, and Herpesvirus B). (2019). Available at: https://www.cdc.gov/herpesbvirus/index.html.

[77] Wang W Qi W Liu J Du H Zhao L Zheng Y. First human infection case of monkey B virus identified in China, 2021. China CDC Wkly (2021) 3(29):632–3. doi: 10.46234/ccdcw2021.154 PMC8393052 34594951

[78] Holmes GP Hilliard JK Klontz KC Rupert AH Schindler CM Parrish E. B virus (Herpesvirus simiae) infection in humans: epidemiologic investigation of a cluster. Ann Internal Med (1990) 112(11):833–9. doi: 10.7326/0003-4819-112-11-833 2160783

[79] Fierer J Bazely P Braude AI. Herpes B virus encephalomyelitis presenting as ophthalmic zoster. A possible latent infection reactivated. Ann Internal Med (1973) 79(2):225–8. doi: 10.7326/0003-4819-79-2-225 4125441

[80] Calvo CM Friedlander S Hilliard J Swarts R Nielsen J Dhindsa H. Case report: reactivation of latent B virus (Macacine herpesvirus 1) presenting as bilateral uveitis, retinal vasculitis and necrotizing herpetic retinitis. Invest Ophthalmol Visual Sci (2011) 52(14):2975–. Available at: https://iovs.arvojournals.org/article.aspx?articleid=2354377.

[81] Oya C Ochiai Y Taniuchi Y Takano T Fujima A Ueda F. Prevalence of herpes B virus genome in the trigeminal ganglia of seropositive cynomolgus macaques. Lab Anim (2008) 42(1):99–103. doi: 10.1258/la.2007.006031 18348771

[82] Davenport DS Johnson DR Holmes GP Jewett DA Ross SC Hilliard JK. Diagnosis and management of human B virus (Herpesvirus simiae) infections in Michigan. Clin Infect Dis an Off Publ Infect Dis Soc America (1994) 19(1):33–41. doi: 10.1093/clinids/19.1.33 7948555

[83] Meechan PJ Potts J. Biosafety in microbiological and biomedical laboratories. (2020). Available at: https://www.cdc.gov/labs/BMBL.html.

[84] Hilliard JK Eberle R Lipper SL Munoz RM Weiss SA. Herpesvirus simiae (B virus): replication of the virus and identification of viral polypeptides in infected cells. Arch Virol (1987) 93(3-4):185–98. doi: 10.1007/bf01310973 3030236

[85] Daniel MD Garcia FG Melendez LV Hunt RD O'Connor J Silva D. Multiple herpesvirus simiae isolation from a rhesus monkey which died of cerebral infarction. Lab Anim Sci (1975) 25(3):303–8. 167229

[86] Vizoso AD. Heterogeneity in herpes simiae (B virus) and some antigenic relationships in the herpes group. Br J Exp Pathol (1974) 55(5):471–7. PMC2072682 4375486

[87] Heberling RL Kalter SS. A dot-immunobinding assay on nitrocellulose with psoralen inactivated herpesvirus simiae (B virus). Lab Anim Sci (1987) 37(3):304–8. 3039249

[88] Reissig M Melnick JL. The cellular changes produced in tissue cultures by herpes B virus correlated with the concurrent multiplication of the virus. J Exp Med (1955) 101(3):341–52. doi: 10.1084/jem.101.3.341 PMC2136472 13233456

[89] Scinicariello F Eberle R Hilliard JK. Rapid detection of B virus (Herpesvirus simiae) DNA by polymerase chain reaction. J Infect Dis (1993) 168(3):747–50. doi: 10.1093/infdis/168.3.747 8394866

[90] Slomka MJ Brown DW Clewley JP Bennett AM Harrington L Kelly DC. Polymerase chain reaction for detection of herpesvirus simiae (B virus) in clinical specimens. Arch Virol (1993) 131(1-2):89–9. doi: 10.1007/bf01379082 8392323

[91] Black DH Eberle R. Detection and differentiation of primate alpha-herpesviruses by Pcr. J Vet Diagn Invest Off Publ Am Assoc Vet Lab Diagnosticians Inc (1997) 9(3):225–31. doi: 10.1177/104063879700900301 9249159

[92] Hirano M Nakamura S Mitsunaga F Okada M Shirahama S Eberle R. One-step Pcr to distinguish B virus from related primate alphaherpesviruses. Clin Diagn Lab Immunol (2002) 9(3):716–9. doi: 10.1128/cdli.9.3.716-719.2002 PMC119990 11986284

[93] Huff JL Eberle R Capitanio J Zhou SS Barry PA. Differential detection of B virus and rhesus cytomegalovirus in rhesus macaques. J Gen Virol (2003) 84(Pt 1):83–92. doi: 10.1099/vir.0.18808-0 12533703

[94] Perelygina L Patrusheva I Manes N Wildes MJ Krug P Hilliard JK. Quantitative real-time Pcr for detection of monkey B virus (Cercopithecine herpesvirus 1) in clinical samples. J Virol Methods (2003) 109(2):245–51. doi: 10.1016/s0166-0934(03)00078-8 12711069

[95] Miranda MB Handermann M Darai G. DNA polymerase gene locus of cercopithecine herpesvirus 1 is a suitable target for specific and rapid identification of viral infection by Pcr technology. Virus Genes (2005) 30(3):307–22. doi: 10.1007/s11262-004-6773-0 15830148

[96] Zeng Y Zhao Y Ren X Zhou X Zhang C Wan Z. Rapid detection of monkeypox virus and monkey B virus by a multiplex loop-mediated isothermal amplification assay. J Infect (2023) 86(4):e114–e6. doi: 10.1016/j.jinf.2023.02.003 PMC9924052 36792036

[97] Poggio P Songia P Vavassori C Ricci V Banfi C Barbieri SS. Digital PCR for high sensitivity viral detection in false-negative Sars-Cov-2 patients. Sci Rep (2021) 11(1):4310. doi: 10.1038/s41598-021-83723-x 33619321 PMC7900100

[98] Liang Y Lin H Zou L Zhao J Li B Wang H. Crispr-Cas12a-based detection for the major Sars-Cov-2 variants of concern. Microbiol Spectr (2021) 9(3):e0101721. doi: 10.1128/Spectrum.01017-21 34787487 PMC8597640

[99] Yin H Tong Z Shen C Xu X Ma H Wu Z. Micro-Pcr chip-based multifunctional ultrafast Sars-Cov-2 detection platform. Lab Chip (2022) 22(14):2671–81. doi: 10.1039/d2lc00101b 35543190

[100] Heberling RL Kalter SS. Rapid dot-immunobinding assay on nitrocellulose for viral antibodies. J Clin Microbiol (1986) 23(1):109–13. doi: 10.1128/jcm.23.1.109-113.1986 PMC268581 3009525

[101] Ohsawa K Lehenbauer TW Eberle R. Herpesvirus Papio 2: alternative antigen for use in monkey B virus diagnostic assays. Lab Anim Sci (1999) 49(6):605–16. 10638495

[102] Takano J Narita T Fujimoto K Mukai R Yamada A. Detection of B virus infection in cynomolgus monkeys by Elisa using simian agent 8 as alternative antigen. Exp Anim (2001) 50(4):345–7. doi: 10.1538/expanim.50.345 11515100

[103] Katz D Shi W Wildes MJ Krug PW Hilliard JK. Reassessing the detection of B-virus-specific serum antibodies. Comp Med (2012) 62(6):516–26. PMC3527757 23561886

[104] Katz D Hilliard JK Eberle R Lipper SL. Elisa for detection of group-common and virus-specific antibodies in human and simian sera induced by herpes simplex and related simian viruses. J Virol Methods (1986) 14(2):99–109. doi: 10.1016/0166-0934(86)90040-6 3021805

[105] Norcott JP Brown DW. Competitive radioimmunoassay to detect antibodies to herpes B virus and Sa8 virus. J Clin Microbiol (1993) 31(4):931–5. doi: 10.1128/jcm.31.4.931-935.1993 PMC263589 8385154

[106] Tanabayashi K Mukai R Yamada A. Detection of B virus antibody in monkey sera using glycoprotein D expressed in mammalian cells. J Clin Microbiol (2001) 39(9):3025–30. doi: 10.1128/JCM.39.9.3025-3030.2001 PMC88291 11526123

[107] Perelygina L Patrusheva I Hombaiah S Zurkuhlen H Wildes MJ Patrushev N. Production of herpes B virus recombinant glycoproteins and evaluation of their diagnostic potential. J Clin Microbiol (2005) 43(2):620–8. doi: 10.1128/JCM.43.2.620-628.2005 PMC548098 15695655

[108] Fujima A Ochiai Y Saito A Omori Y Noda A Kazuyama Y. Discrimination of antibody to herpes B virus from antibody to herpes simplex virus types 1 and 2 in human and macaque sera. J Clin Microbiol (2008) 46(1):56–61. doi: 10.1128/jcm.00342-07 17989200 PMC2224259

[109] Katz D Shi W Patrusheva I Perelygina L Gowda MS Krug PW. An automated Elisa using recombinant antigens for serologic diagnosis of B virus infections in macaques. Comp Med (2012) 62(6):527–34. PMC3527758 23561887

[110] Perelygina L Zurkuhlen H Patrusheva I Hilliard JK. Identification of a herpes B virus-specific glycoprotein D immunodominant epitope recognized by natural and foreign hosts. J Infect Dis (2002) 186(4):453–61. doi: 10.1086/341834 12195371

[111] Hotop SK Abd El Wahed A Beutling U Jentsch D Motzkus D Frank R. Multiple antibody targets on herpes B glycoproteins B and D identified by screening sera of infected rhesus macaques with peptide microarrays. PloS One (2014) 9(1):e86857. doi: 10.1371/journal.pone.0086857 24497986 PMC3908960

[112] Hotop SK Abd El Wahed A Beutling U Czerny F Sievers C Diederichsen U. Serological analysis of herpes B virus at individual epitope resolution: from two-dimensional peptide arrays to multiplex bead flow assays. Anal Chem (2019) 91(17):11030–7. doi: 10.1021/acs.analchem.9b01291 31365232

[113] Cropper LM Lees DN Patt R Sharp IR Brown D. Monoclonal antibodies for the identification of herpesvirus simiae (B virus). Arch Virol (1992) 123(3-4):267–77. doi: 10.1007/bf01317263 1314049

[114] Blewett EL Black D Eberle R. Characterization of virus-specific and cross-reactive monoclonal antibodies to herpesvirus simiae (B virus). J Gen Virol (1996) 77(Pt 11):2787–93. doi: 10.1099/0022-1317-77-11-2787 8922473

[115] Katz D Shi W Gowda MS Vasireddi M Patrusheva I Seoh H-K. Identification of unique B virus (Macacine herpesvirus 1) epitopes of zoonotic and macaque isolates using monoclonal antibodies. PloS One (2017) 12(8):e0182355. doi: 10.1371/journal.pone.0182355 28783746 PMC5544422

[116] James C Harfouche M Welton NJ Turner KM Abu-Raddad LJ Gottlieb SL. Herpes simplex virus: global infection prevalence and incidence estimates, 2016. Bull World Health Organ (2020) 98(5):315–29. doi: 10.2471/blt.19.237149 PMC7265941 32514197

[117] Ward JA Hilliard JK. Herpes B virus-specific pathogen-free breeding colonies of macaques: serologic test results and the B-virus status of the macaque. Contemp Topics Lab Anim Sci (2002) 41(4):36–41. 12109895

[118] Weigler BJ Scinicariello F Hilliard JK. Risk of venereal B virus (Cercopithecine herpesvirus 1) transmission in rhesus monkeys using molecular epidemiology. J Infect Dis (1995) 171(5):1139–43. doi: 10.1093/infdis/171.5.1139 7751688

[119] Ma H Zeng W He H Zhao D Jiang D Zhou P. Serum Iga, Igm, and Igg responses in Covid-19. Cell Mol Immunol (2020) 17(7):773–5. doi: 10.1038/s41423-020-0474-z PMC7331804 32467617

[120] Infantino M Manfredi M Grossi V Lari B Fabbri S Benucci M. Closing the serological gap in the diagnostic testing for Covid-19: the value of anti-Sars-Cov-2 Iga antibodies. J Med Virol (2021) 93(3):1436–42. doi: 10.1002/jmv.26422 PMC7436746 32790181

[121] Lawandi A Danner RL. Antibody tests have higher sensitivity at ≥15 days after symptom onset and 99% specificity for detecting SARS-CoV-2. Ann Intern Med (2020) 173(10):JC57. doi: 10.7326/ACPJ202011170-057 33197346 PMC10807479

[122] Focher F Lossani A Verri A Spadari S Maioli A Gambino JJ. Sensitivity of monkey B virus (Cercopithecine herpesvirus 1) to antiviral drugs: role of thymidine kinase in antiviral activities of substrate analogs and acyclonucleosides. Antimicrob Agents Chemother (2007) 51(6):2028–34. doi: 10.1128/aac.01284-06 PMC1891389 17438061

[123] Boulter EA Thornton B Bauer DJ Bye A. Successful treatment of experimental B virus (Herpesvirus simiae) infection with acyclovir. Br Med J (1980) 280:681–3. doi: 10.1136/bmj.280.6215.681 PMC1600772 6244873

[124] Zwartouw HT Humphreys CR Collins P. Oral chemotherapy of fatal B virus (Herpesvirus simiae) infection. Antiviral Res (1989) 11(5-6):275–83. doi: 10.1016/0166-3542(89)90037-5 2552914

[125] Holmes GP Chapman LE Stewart JA Straus SE Hilliard JK Davenport DS. Guidelines for the prevention and treatment of B-virus infections in exposed persons. The B virus working group. Clin Infect Dis an Off Publ Infect Dis Soc America (1995) 20(2):421–39. doi: 10.1093/clinids/20.2.421 7742451

[126] Scinicariello F English WJ Hilliard J. Identification by Pcr of meningitis caused by herpes B virus. Lancet (London England) (1993) 341(8861):1660–1. doi: 10.1016/0140-6736(93)90791-e 8100019

[127] Artenstein AW Hicks CB Goodwin BS Jr. Hilliard JK. Human infection with B virus following a needlestick injury. Rev Infect Dis (1991) 13(2):288–91. doi: 10.1093/clinids/13.2.288 1645881

[128] Bryan BL Espana CD Emmons RW Vijayan N Hoeprich PD. Recovery from encephalomyelitis caused by herpesvirus simiae. Report of a case. Arch Internal Med (1975) 135(6):868–70. doi: 10.1001/archinte.1975.00330060112017 165794

[129] Nanda M Curtin VT Hilliard JK Bernstein ND Dix RD. Ocular histopathologic findings in a case of human herpes B virus infection. Arch Ophthalmol (Chicago Ill 1960) (1990) 108(5):713–6. doi: 10.1001/archopht.1990.01070070099044 2159276

[130] Melnick JL Banker DD. Isolation of B virus (Herpes group) from the central nervous system of a rhesus monkey. J Exp Med (1954) 100(2):181–94. doi: 10.1084/jem.100.2.181 PMC2136366 13286422

[131] Cabasso VJ Chappell WA Avampato JE Bittle JL. Correlation of B virus and herpes simplex virus antibodies in human sera. J Lab Clin Med (1967) 70(1):170–8. 4290842

[132] Boulter EA Zwartouw HT Thornton B. Postexposure immunoprophylaxis against B virus (Herpesvirus simiae) infection. Br Med J (Clinical Res ed) (1981) 283(6305):1495–7. doi: 10.1136/bmj.283.6305.1495 PMC1507841 6275934

[133] Breen GE Lamb SG Otaki AT. Monkey-bite encephalomyelitis; report of a case; with recovery. Br Med J (1958) 2(5087):22–3. doi: 10.1136/bmj.2.5087.22 PMC2025841 13546633

[134] Hummeler K Davidson WL Henle W Laboccetta AC Ruch HG. Encephalomyelitis due to infection with herpesvirus simiae (Herpes B virus); a report of two fatal, laboratory-acquired cases. New Engl J Med (1959) 261(2):64–8. doi: 10.1056/nejm195907092610203 13666979

[135] Vizoso AD. Latency of herpes simiae (B virus) in rabbits. Br J Exp Pathol (1975) 56(6):489–94. PMC2072794 177039

[136] Hull RN Nash JC. Immunization against B virus infection. I. Preparation of an experimental vaccine. Am J Hyg (1960) 71:15–28. doi: 10.1093/oxfordjournals.aje.a120086 14403944

[137] Hull RN Peck FB Jr. Ward TG Nash JC. Immunization against B virus infection. Ii. Further laboratory and clinical studies with an experimental vaccine. Am J Hyg (1962) 76:239–51. 13955640

[138] Hull RN. B virus vaccine. Lab Anim Sci (1971) 21(6):1068–71. 4331930

[139] Bennett AM Slomka MJ Brown DW Lloyd G Mackett M. Protection against Herpes B virus infection in rabbits with a recombinant vaccinia virus expressing glycoprotein D. J Med Virol (1999) 57(1):47–56. doi: 10.1002/(sici)1096-9071(199901)57:1<47::aid-jmv7>3.0.co;2-v 9890421

[140] Loomis-Huff JE Eberle R Lockridge KM Rhodes G Barry PA. Immunogenicity of a DNA vaccine against herpes B virus in mice and rhesus macaques. Vaccine (2001) 19(32):4865–73. doi: 10.1016/s0264-410x(01)00232-8 11535340

[141] Hirano M Nakamura S Mitsunaga F Okada M Shimizu K Ueda M. Efficacy of a B virus Gd DNA vaccine for induction of humoral and cellular immune responses in Japanese macaques. Vaccine (2002) 20(19-20):2523–32. doi: 10.1016/s0264-410x(02)00175-5 12057608

[142] Sabin AB. Studies on the B virus. I: the immunological identity of a virus isolated from a human case of ascending myelitis associated with visceral necrosis. Br J Exp Pathol (1934) 15(4):248–68.

[143] Black FL Melnick JL. Microepidemiology of poliomyelitis and herpes-B infections: spread of the viruses within tissue cultures. J Immu (1955) 74 3:236–42. 14354206

[144] Keadle TL Laycock KA Miller JK Hook KK Fenoglio ED Francotte M. Efficacy of a recombinant glycoprotein D subunit vaccine on the development of primary and recurrent ocular infection with herpes simplex virus type 1 in mice. J Infect Dis (1997) 176(2):331–8. doi: 10.1086/514049 9237697

[145] Belshe RB Leone PA Bernstein DI Wald A Levin MJ Stapleton JT. Efficacy results of a trial of a herpes simplex vaccine. New Engl J Med (2012) 366(1):34–43. doi: 10.1056/NEJMoa1103151 22216840 PMC3287348

[146] Cortesi R Ravani L Rinaldi F Marconi P Drechsler M Manservigi M. Intranasal immunization in mice with non-ionic surfactants vesicles containing Hsv immunogens: A preliminary study as possible vaccine against genital herpes. Int J Pharm (2013) 440(2):229–37. doi: 10.1016/j.ijpharm.2012.06.042 22743007

[147] Chiuppesi F Vannucci L De Luca A Lai M Matteoli B Freer G. A lentiviral vector-based, herpes simplex virus 1 (Hsv-1) glycoprotein B vaccine affords cross-protection against Hsv-1 and Hsv-2 genital infections. J Virol (2012) 86(12):6563–74. doi: 10.1128/jvi.00302-12 PMC3393530 22491465

[148] Posavad CM Koelle DM Corey L. Tipping the scales of herpes simplex virus reactivation: the important responses are local. Nat Med (1998) 4(4):381–2. doi: 10.1038/nm0498-381 9546773

[149] Koelle DM Posavad CM Barnum G Johnson ML Frank JM Corey L. Clearance of Hsv-2 from recurrent genital lesions correlates with infiltration of Hsv-specific cytotoxic T lymphocytes. J Clin Invest (1998) 101 7:1500–8. doi: 10.1172/JCI1758 PMC508728 9525993

[150] Verjans GM Hintzen RQ van Dun JM Poot A Milikan JC Laman JD. Selective retention of herpes simplex virus-specific T cells in latently infected human trigeminal ganglia. Proc Natl Acad Sci United States America (2007) 104(9):3496–501. doi: 10.1073/pnas.0610847104 PMC1805572 17360672

[151] Schiffer JT Abu-Raddad L Mark KE Zhu J Selke S Koelle DM. Mucosal host immune response predicts the severity and duration of herpes simplex virus-2 genital tract shedding episodes. Proc Natl Acad Sci United States America (2010) 107(44):18973–8. doi: 10.1073/pnas.1006614107 PMC2973882 20956313

[152] Prichard MN Kaiwar R Jackman WT Quenelle DC Collins DJ Kern ER. Evaluation of Ad472, a live attenuated recombinant herpes simplex virus type 2 vaccine in guinea pigs. Vaccine (2005) 23(46-47):5424–31. doi: 10.1016/j.vaccine.2005.02.028 PMC2718572 15950327

[153] Bernstein DI Cardin RD Smith GA Pickard GE Sollars PJ Dixon DA. The R2 non-neuroinvasive Hsv-1 vaccine affords protection from genital Hsv-2 infections in a guinea pig model. NPJ Vaccines (2020) 5(1):104. doi: 10.1038/s41541-020-00254-8 33298966 PMC7648054

[154] Awasthi S Lubinski JM Shaw CE Barrett SM Cai M Wang F. Immunization with a vaccine combining herpes simplex virus 2 (Hsv-2) glycoprotein C (Gc) and Gd subunits improves the protection of dorsal root ganglia in mice and reduces the frequency of recurrent vaginal shedding of Hsv-2 DNA in guinea pigs compared to immunization with Gd alone. J Virol (2011) 85(20):10472–86. doi: 10.1128/jvi.00849-11 PMC3187515 21813597

[155] Dutton JL Li B Woo WP Marshak JO Xu Y Huang ML. A novel DNA vaccine technology conveying protection against a lethal herpes simplex viral challenge in mice. PloS One (2013) 8(10):e76407. doi: 10.1371/journal.pone.0076407 24098493 PMC3789751

[156] Sahin U Muik A Vogler I Derhovanessian E Kranz LM Vormehr M. Bnt162b2 vaccine induces neutralizing antibodies and poly-specific T cells in humans. Nature (2021) 595(7868):572–7. doi: 10.1038/s41586-021-03653-6 34044428

[157] Baden LR El Sahly HM Essink B Kotloff K Frey S Novak R. Efficacy and safety of the Mrna-1273 Sars-Cov-2 vaccine. New Engl J Med (2021) 384(5):403–16. doi: 10.1056/NEJMoa2035389 PMC7787219 33378609

[158] Tang R Zheng H Wang BS Gou JB Guo XL Chen XQ. Safety and immunogenicity of aerosolised Ad5-Ncov, intramuscular Ad5-Ncov, or inactivated Covid-19 vaccine Coronavac given as the second booster following three doses of Coronavac: A multicentre, open-label, phase 4, randomised trial. Lancet Respir Med (2023) 11(7):613–23. doi: 10.1016/s2213-2600(23)00049-8 PMC9991083 36898400

